# Evolution of Linked Avirulence Effectors in *Leptosphaeria maculans* Is Affected by Genomic Environment and Exposure to Resistance Genes in Host Plants

**DOI:** 10.1371/journal.ppat.1001180

**Published:** 2010-11-04

**Authors:** Angela P. Van de Wouw, Anton J. Cozijnsen, James K. Hane, Patrick C. Brunner, Bruce A. McDonald, Richard P. Oliver, Barbara J. Howlett

**Affiliations:** 1 School of Botany, the University of Melbourne, Victoria, Australia; 2 Australian Centre for Necrotrophic Fungal Pathogens, Curtin University, Bentley, Western Australia, Australia; 3 Plant Pathology Group, Institute of Integrative Biology, ETH Zurich, Zurich, Switzerland; University of California San Francisco, United States of America

## Abstract

*Brassica napus* (canola) cultivars and isolates of the blackleg fungus, *Leptosphaeria maculans* interact in a ‘gene for gene’ manner whereby plant resistance (*R*) genes are complementary to pathogen avirulence (*Avr*) genes. Avirulence genes encode proteins that belong to a class of pathogen molecules known as effectors, which includes small secreted proteins that play a role in disease. In Australia in 2003 canola cultivars with the *Rlm1* resistance gene suffered a breakdown of disease resistance, resulting in severe yield losses. This was associated with a large increase in the frequency of virulence alleles of the complementary avirulence gene, *AvrLm1*, in fungal populations. Surprisingly, the frequency of virulence alleles of *AvrLm6* (complementary to *Rlm6*) also increased dramatically, even though the cultivars did not contain *Rlm6*. In the *L. maculans* genome, *AvrLm1* and *AvrLm6* are linked along with five other genes in a region interspersed with transposable elements that have been degenerated by Repeat-Induced Point (RIP) mutations. Analyses of 295 Australian isolates showed deletions, RIP mutations and/or non-RIP derived amino acid substitutions in the predicted proteins encoded by these seven genes. The degree of RIP mutations within single copy sequences in this region was proportional to their proximity to the degenerated transposable elements. The RIP alleles were monophyletic and were present only in isolates collected after resistance conferred by *Rlm1* broke down, whereas deletion alleles belonged to several polyphyletic lineages and were present before and after the resistance breakdown. Thus, genomic environment and exposure to resistance genes in *B. napus* has affected the evolution of these linked avirulence genes in *L. maculans*.

## Author Summary

The fungus *Leptosphaeria maculans* causes blackleg, the major disease of canola worldwide. Populations of this fungus rapidly adapt to selection pressures such as the extensive sowing of canola with particular disease resistance genes. This can lead to a breakdown of resistance and severe economic losses. We describe mutations in key fungal genes involved in the interaction with canola, and we report the first large scale study of evolutionary processes affecting such genes in any fungal plant pathogen. We relate these changes to the genomic environment of these genes and to the breakdown of disease resistance in canola.

## Introduction

The fungus *Leptosphaeria maculans* causes blackleg (phoma stem canker) and is the major disease of *Brassica napus* (canola) worldwide [1]. The major source of inoculum is wind-borne ascospores, which are released from sexual fruiting bodies on infected stubble (crop residue) of previous crops and can be transmitted several kilometres. This fungus has a ‘gene for gene’ interaction with its host (canola) such that pathogen avirulence alleles render the pathogen unable to attack host genotypes with the corresponding resistance genes [2].

Twelve genes conferring resistance to *L. maculans* (*Rlm1-9, LepR1-3*) have been identified from *Brassica* species [3], [4]. Nine of these genes have been mapped but none have yet been cloned [5], [6]. Of the corresponding avirulence genes in *L. maculans*, seven have been mapped to two gene clusters, *AvrLm1-2-6* and *AvrLm3-4-7-9*, located on separate *L. maculans* chromosomes [7]. Three genes, *AvrLm1*, *AvrLm6* and *AvrLm4-7*, have been cloned and characterised [8], [9], [10]. Avirulence proteins belong to a class of molecules called effectors. Effectors are small molecules or proteins produced by the pathogen that alter host-cell structure and function, facilitate infection (for example, toxins) and/or induce defence responses (for example, avirulence proteins), and are generally essential for disease progression [11]. Many effectors are small, secreted proteins (SSPs), which are cysteine-rich, share no sequence similarity with genes from other species, are often highly polymorphic between isolates of a single species and are expressed highly *in planta* [12]. The AvrLm1, AvrLm4-7 and AvrLm6 proteins of *L. maculans* are effector molecules with an avirulence function. *AvrLm6* and *AvrLm4-7* encode SSPs with six and eight cysteine residues, respectively, whilst the *AvrLm1* protein, which is also a SSP, has only one cysteine [8], [9], [10].

*Leptosphaeria maculans* undergoes sexual recombination prolifically and populations can rapidly adapt to selection pressures imposed by the host such as exposure to resistance conferred by single or major genes. This situation increases the frequency of virulent isolates and can cause resistance to break down, often resulting in severe yield losses [13]. Three examples of this breakdown are discussed below, the most dramatic one occurring in Australia in 2003.

Prior to 2000 the Australian canola industry relied on ‘polygenic’ cultivars with multiple resistance genes. Yield losses were generally low. In 2000, cultivars with major gene resistance, termed ‘sylvestris’ resistance, were released commercially and grown extensively in some areas of Australia. These cultivars were derived from a synthetic *B. napus* line produced by crossing the two progenitor species, *B. oleracea* subsp. *alboglabra* and an accession of *B. rapa* subsp. *sylvestris* that had a high level of resistance to *L. maculans* [14]. For several years these cultivars showed little or no disease but in 2003, resistance failed, resulting in up to 90% yield losses in the Eyre Peninsula, South Australia, costing the industry between $5–10 million AUD [15], [16]. These cultivars contained resistance genes *Rlm1* and *RlmS*, suggesting that both sources of resistance failed simultaneously [17]. After 2004, these cultivars were withdrawn from sale although they were still grown in yield trial sites around Australia. A similar but less dramatic situation occurred in France when resistance conferred by *Rlm1* was rendered ineffective within five years of commercial release of *Rlm1-*containing cultivars [13], [18]. Another breakdown of resistance was observed in field trial experiments in France that had been designed to assess the durability of a resistance gene *Rlm6*. In these experiments *B. napus* lines containing *Rlm6* were sown into *L. maculans*-infected stubble of a *Rlm6*–containing line over a four year period [19]. After three years of this contrived selection, the frequency of virulence in fungal populations towards *Rlm6* was so high that this resistance was rendered ineffective and the lines suffered extremely high levels of disease.

The three avirulence genes, *AvrLm1*, *AvrLm6* and *AvrLm4-7*, cloned from *L. maculans* are located within AT-rich, gene-poor regions that are riddled with degenerated copies of transposable elements [8], [9], [10]. These transposable elements appear to have been inactivated via repeat-induced point (RIP) mutations [20]. RIP is an ascomycete-specific process that alters the sequence of multicopy DNA. Nucleotide changes CpA to TpA and TpG to TpA are conferred during meiosis, often generating stop codons, thereby inactivating genes [21]. Additionally, RIP mutations have been inferred bioinformatically in various transposable elements throughout the *L. maculans* genome and in *AvrLm6* [22]. *AvrLm1* and *AvrLm6* are genetically linked and different types of mutations leading to virulence have been reported. The entire *AvrLm1* locus was deleted in 285 of 290 (98%) isolates that were virulent towards *Rlm1* [20]. Fudal et al. characterised the *AvrLm6* locus in a different set of 105 isolates, most of which were cultured from *Rlm6*–containing lines during the field trial in France described above [22], [23]. Deletions and RIP were responsible for virulence in 45 (66%) and 17 (24%) isolates, respectively, that were virulent towards the *Rlm6* gene [22].

In this paper we relate changes in the types and frequencies of mutations in genes including *AvrLm1* and *AvrLm6* to the selection pressure imposed by extensive regional sowing of *B. napus* cultivars with sylvestris resistance.

## Results

### Virulence of Australian isolates of *L. maculans* and analysis of mutations at *AvrLm1* and *AvrLm6*

In preliminary experiments to see if the breakdown of ‘sylvestris resistance’ in *B. napus* seen in the field [15] correlated with changes in frequency of virulence towards *Rlm1* in individual *L. maculans* isolates, 11 isolates collected prior to the breakdown (before 2004) and 12 isolates collected after the breakdown (2004 onwards) were screened for virulence on *B. napus* cultivars Q2 (with *Rlm3*) and Columbus (*Rlm1, Rlm3*). Cotyledons of 14 day old plants were inoculated with individual isolates and symptoms were scored 17 days later. All isolates were virulent on the susceptible control, cv. Q2. Ten of the 11 isolates collected prior to 2004 were avirulent on the *Rlm1*-containing cultivar, Columbus. However, seven of ten isolates collected from 2004 onwards were virulent towards *Rlm1* (Table 1). A subset of these isolates was inoculated onto the *B. napus* cultivar Aurea (*Rlm6*). Of seven of the 11 isolates collected prior to 2004, only one was virulent towards *Rlm6*. However, of 12 isolates collected from 2004 onwards, eight were virulent towards *Rlm6* (Table 1). These results suggested that there was a significant change in frequencies of virulent alleles of *AvrLm1* and *AvrLm6* associated with breakdown of ‘sylvestris resistance’. These isolates were then genotyped at the *AvrLm1*, *AvrLm6* and mating type loci using PCR-based screening [20], [22], [24] and as expected, the virulence phenotypes corresponded with *avrLm1* and /or *avrLm6* genotypes (Table 1).

**Table 1:** Pathogenicity of Australian isolates of *Leptosphaeria maculans* on cotyledons of *Brassica napus* cultivars Q2 and Columbus and *B. juncea* cv. Aurea.

Isolate	Cultivar						Genotype^a^	
	Q2 (*Rlm3*)		Columbus (*Rlm1, Rlm3*)		Aurea (*Rlm5, Rlm6*)			
	Path. score	Phenotype	Path. score	Phenotype	Path. score	Phenotype	*AvrLm1* allele	*AvrLm6* allele
Pre sylvestris breakdown (before 2004)								
LM535	6.5	Vir	2.6	Avir	Not tested		0	0
1317	6.4	Vir	1.9	Avir	Not tested		0	0
LM752	4.5	Vir	1.2	Avir	1.5	Avir	0	1
LM526	6.5	Vir	1.4	Avir	2.3	Avir	0	1
LM641	6.6	Vir	1.4	Avir	Not tested		0	1
LM749	6.5	Vir	1.8	Avir	1.0	Avir	0	1
GA2	7.0	Vir	1.5	Avir	1.6	Avir	0	3
IBCN18	7.0	Vir	2.3	Avir	6.7	Vir	1	del
V4	7.1	Vir	1.3	Avir	Not tested		1	del
LM691	7.0	Vir	1.1	Avir	1.2	Avir	3	0
IBCN17	6.7	Vir	6.6	Vir	1.6	Avir	del	0
Post sylvestris breakdown (2004 onwards)								
04P017	6.2	Vir	1.3	Avir	1.5	Avir	0	1
04P042	5.7	Vir	1.2	Avir	1.3	Avir	0	1
04S005	7.0	Vir	7.8	Vir	1.8	Avir	del	0
04S014	7.0	Vir	7.2	Vir	1.6	Avir	del	1
05P032	7.0	Vir	6.5	Vir	6.4	Vir	del	2
05P033	6.5	Vir	6.7	Vir	6.0	Vir	del	2
05P034	7.0	Vir	6.7	Vir	5.6	Vir	del	2
06S013	4.8	Vir	7.1	Vir	4.1	Vir	del	6
06S039	5.8	Vir	6.9	Vir	4.6	Vir	del	8
06P042	7.0	Vir	1.6	Avir	3.6	Vir	0	9
06P039	7.0	Vir	1.1	Avir	4.1	Vir	0	9
06P040	7.0	Vir	1.3	Avir	4.8	Vir	1	*del*

Cotyledons of 14 day old seedlings were infected with spores of individual isolates. Mean pathogenicity scores (Path. score) were determined by assessing 40 inoculation sites at 17 dpi. Isolates with Path. scores ≤3.9 are classified as avirulent whilst those with Path. scores ≥4.0 are classified as virulent.

a Alleles as described in Table 2.

Changes in allele frequencies were then examined in a total of 295 Australian isolates. Of these 137 were collected between 1987 and 2003, prior to the breakdown of sylvestris resistance, whilst the remaining 158 isolates were collected between 2004 and 2008, after the resistance breakdown (Table S1). These isolates were collected from stubble of a range of canola cultivars with different resistance genes. One third of the isolates had a deletion of *AvrLm1* (Table 2), whilst 63% had the allele of isolate v23.1.3, whose genome has been sequenced. Alleles of this isolate hereafter are referred to as wild type alleles (e.g. *AvrLm1*-*0*). As expected, isolates with *AvrLm1*-*0* were avirulent towards *Rlm1* (Tables 1 and 2). The remaining eight isolates comprised four alleles with coding sequence changes conferring non-synonymous substitutions (I^125^K and/or H^155^Y). Isolates harbouring these alleles were avirulent on the *Rlm1*- containing *B. napus* line (Table 1). Thirteen alleles of *AvrLm6* were detected (Table 2). *AvrLm6* was deleted in 20% of isolates, thus conferring virulence towards *Rlm6*, whilst 24% had the allele of the sequenced isolate and conferred avirulence towards *Rlm6* (Tables 1 and 2). Other isolates virulent towards the *Rlm6*-containing cultivar had alleles with stop codons conferred by base changes reminiscent of RIP mutation. Accordingly, allele sequences were analysed by RIPCAL, a software tool that visualises the physical distribution of RIP mutation and reports a RIP dominance score indicating the degree of RIP in each sequence. Sequences with RIP dominance scores >1 are highly RIP-affected having a high proportion of CpA to TpA, or TpG to TpC RIP mutations [25]. RIPCAL analysis did not detect RIP in any *AvrLm1* alleles, but detected RIP in seven *AvrLm6* alleles. These RIP mutations resulted in numerous non-synonymous changes as well as premature stop codons (between 4 and 6), which were within all RIP-affected alleles (Table 2). Southern hybridisation results suggest that there is only a single copy of the *AvrLm6* locus within isolates harbouring the RIP alleles (Figure S1). The remaining alleles of *AvrLm6* (*AvrLm6-1, -2,-3* and *-4*) harboured single or few nucleotide changes leading to synonymous or amino acid substitutions (G^123^C, K^127^E, F^54^L) compared to isolate v23.1.3 (*AvrLm6-0*). These amino acid substitutions were generated via non-RIP like mutations (henceforth referred to as non-RIP amino acid substitutions). Isolates harbouring *AvrLm6-1, AvrLm6-3* or *AvrLm6-4* alleles were avirulent towards cv. Aurea, whilst the four isolates harbouring the *AvrLm6-2* allele (G^123^C) were virulent (Table 1).

**Table 2:** Alleles of *AvrLm1*, *AvrLm6, LmCys1* and *LmCys2* in 295 Australian isolates of *Leptosphaeria maculans*.

Genea, b	Allele	Isolates (frequency %)	Nucleotide changes^c^				Coding sequence changes^d^	RIP dominance score^e^
			No.	Type				
				CpA to TpA	TpG to TpA	Other		
*AvrLm1*	0	185 (62.8)	0	0	0	0		0
	1	9 (3.0)	1	0	0	1 (T to A)	I^125^K	0
	2	1 (0.3)	2	0	0	2 (T to A, G to T)	I^125^K	0
	3	2 (0.7)	2	1	0	1 (T to A)	I^125^K, H^155^Y	0
	4	1 (0.3)	3	1	0	2 (T to A, G to T)	I^125^K, H^155^Y	0
	del	97 (32.9)	deletion					*NC*
*AvrLm6*	0	70 (23.7)	0	0	0	0		0
	1	134 (45.4)	1	0	1	0	SYN	0
	2	4 (1.4)	2	0	1	1 (G to T)	G^123^C	0
	3	4 (1.4)	2	0	1	1 (A to G)	K^127^E	0
	4	2 (0.7)	3	0	1	2 (T to C, A to G)	F^54^L, K^127^E	0
	5	1 (0.3)	38	10	15	13 (all G to A or C to T)	16 N-S, 5 SC	3.6
	6	1 (0.3)	41	11	13	17 (all G to A or C to T)	22 N-S, 4 SC	3.0
	7	3 (1.0)	42	11	14	17 (all G to A or C to T)	21 N-S, 4 SC	2.8
	8	3 (1.0)	41	14	15	12 (all G to A or C to T)	21 N-S, 6 SC	9.7
	9	3 (1.0)	46	18	12	16 (all G to A or C to T)	18 N-S, 6 SC	7.5
	10	1 (0.3)	42	13	13	16 (all G to A or C to T)	20 N-S, 5 SC	3.3
	11	2 (0.7)	46	13	14	19 (all G to A or C to T)	25 N-S, 5 SC	3.9
	del	67 (22.8)	deletion					*NC*
*LmCys1*	0	52 (17.6)	0	0	0	0		0
	1	238 (80.7)	1	0	0	1 (A to C)	N^121^H	0
	2	2 (0.7)	3	0	0	3 (A to G, A to G, C to G)	SYN, N^121^G, Q^134^E	0
	3	2 (0.7)	4	0	0	4 (A to G, C to G, A to G, A to G)	SYN, T^53^A, N^121^G	0
	4	1 (0.3)	63	32	12	19 (all G to A or C to T)	26 N-S, 18 SC	7.3
*LmCys2*	0	293 (99.3)	0	0	0	0		0
	del	2 (0.7)	deletion					*NC*

a The reference sequences are AM084345 (designated as *AvrLm1-0*), AM2539336 (*AvrLm6-0*) and GU332625 (*LmCys1-0*) and GU332629 (*LmCys2-0*) in isolate v23.1.3 [8], [9].

b Sizes of amplified products were 676 bp for *AvrLm1-0*, 751 bp for *AvrLm6-0*, 667 bp for *LmCys1-0* and 1050 bp for *LmCys2-0*.

c Deletions were confirmed by Southern analysis of selected isolates (Figure S1).

d SYN, synonymous amino acid substitutions; N-S, non-synonymous substitutions; SC, premature stop codons; *NC*  =  Not calculated.

e Allele sequences were analysed by RIPCAL for the presence of RIP mutations [25]. All sequences were compared to the wild-type allele (designated *-0*). RIP dominance scores of >1 are highly RIP-affected, whilst scores of 0 reflect the absence of RIP.

No RIP-affected alleles were present in isolates collected prior to the breakdown of sylvestris resistance. However, the 158 isolates collected after the breakdown had seven *AvrLm6* RIP alleles (frequency of 8.9%) and there was a very large increase in the frequency of deletion alleles of *AvrLm1* (22.6 to 41.8%) and *AvrLm6* (4.4 to 38.7). This was a nine-fold increase in frequency of *avrLm6* (Table S2). Thirty four (11.5%) isolates had deletions of both *AvrLm1* and *AvrLm6* and only one of these isolates was collected prior to the breakdown. All 295 isolates were grouped into four genotypic classes (*AvrLm1, AvrLm6*; *AvrLm1, avrLm6*; *avrLm1, Avrlm6*; *avrLm1, avrlm6*). The frequency of *AvrLm1, AvrLm6* isolates was 73.7% prior to the breakdown, but decreased to 37.3% afterwards (Table 3). Conversely, the frequency of *avrLm1, avrlm6* isolates was only 0.8% prior to the breakdown, but increased to 28.5% afterwards.

**Table 3:** Changes in the frequency of the profile of alleles of *AvrLm1* and *Avrlm6* of *Leptosphaeria maculans* isolates before and after the breakdown of ‘sylvestris resistance’ and in relation to stubble source of isolates.

Genotype	Number of isolates (frequency %)		Number of isolates (frequency %)^a^	
	before 2004	2004 onwards	Polygenic^b^	Sylvestris^b^
*AvrLm1*, *AvrLm6*	101 (73.7)	59 (37.3)	43 (53.8)	0 (0)
*AvrLm1*, *avrLm6*	5 (3.6)	33 (20.9)	25 (31.2)	0 (0)
*avrLm1*, *AvrLm6*	30 (21.9)	21 (13.3)	6 (7.5)	15 (31.3)
*avrLm1*, *avrLm6*	1 (0.8)	45 (28.5)	6 (7.5)	33 (68.7)

a Only isolates collected between 2004 and 2008 were analysed.

b Polygenic and sylvestris refers to the resistance of cultivars from which isolates were cultured.

Nine of the 14 isolates harbouring RIP alleles (64%) had a deletion allele at the *AvrLm1* locus and all these isolates were cultured from stubble of cultivars with sylvestris resistance. Additionally, all four isolates harbouring the virulent *AvrLm6*-2 allele (G^123^C) had a deletion allele at *AvrLm1* (Table S3). When the isolates cultured from 2004 onwards were categorised in terms of the stubble from which they were derived, all those cultured from ‘sylvestris stubble’ had the *avrLm1* allele (Table 3) as expected, due to the presence of *Rlm1* in these cultivars [17]. Conversely, only 15% of isolates cultured from stubble of polygenic cultivars, which do not have *Rlm1* nor *Rlm6*, had the *avrLm1* allele. The frequency of *avrLm6* was 38.7% in isolates cultured from ‘polygenic’ stubble, compared to 68.7% of isolates cultured from ‘sylvestris’ stubble (Table 3). For all comparisons of isolates, there were no significant differences in allele frequencies at the mating type locus. Since the mating-type locus is not under selection pressure, a 1∶1 ratio of each allele suggests that sampling of isolates has been random (data not shown).

### Characteristics of the genomic environment of *AvrLm1* and *AvrLm6*

**Figure 1:**
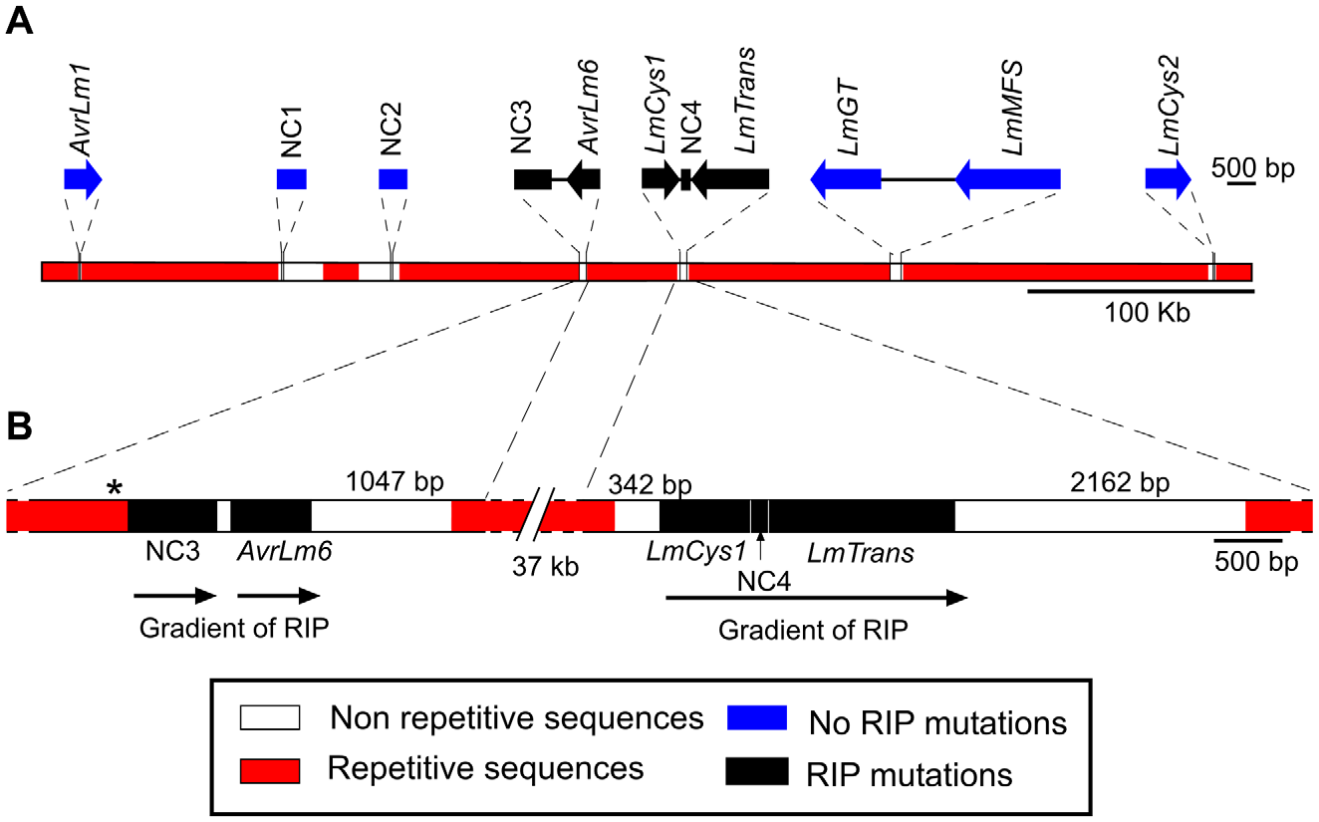
Location of the genes and non-coding, non-repetitive regions analysed from a 520 kb region of the *Leptosphaeria maculans* genome located on a 2.6 Mb chromosome in isolate v23.1.3. (A) Schematic representation of the *AvrLm1-LmCys2* genomic region whereby highly-repetitive sequences with low GC content (red) flank single copy sections with high GC content (white). Four genes encoding small-secreted proteins, *AvrLm1*, *AvrLm6*, *LmCys1* and *LmCys2*, were sequenced from 295 Australian isolates, whilst the remaining three genes and four non-coding, non-repetitive regions (NC1-4) were sequenced in 84 of the 295 isolates. Repeat-induced point (RIP) mutations were detected in *AvrLm6*, *LmCys1*, *LmTrans* and two non-coding, non-repetitive regions (NC3 and NC4) (black). (B) Location of single-copy sequences relative to the flanking repetitive regions. Within each of the two single copy regions(black) analysed, the frequency of RIP alleles and distribution of RIP mutations decreased in a 5′ to 3′gradient (arrows). The single copy region (149 bp) between NC3 and *AvrLm6* was not analysed and so the gradient arrow is discontinuous. * denotes a repeat region (620 bp) directly upstream of NC3 that is highly RIP-affected.

Because of the marked difference in the *AvrLm1* and *AvrLm6* allele frequencies before and after the breakdown of sylvestris resistance, the region flanking these genes was characterised to identify any features that might have influenced allele frequency. A 520 kb AT-rich genomic region bordered by *AvrLm1* and *LmCys2* (Figure 1A) was examined. Part of this region has been described previously in isolate v23.1.3 [8] and includes two additional genes encoding SSPs, *LmCys1* and *LmCys2*. Three other genes, *LmTrans*, *LmGT* and *LmMFS* were present; *LmCys1, LmTrans, LmGT, LmMFS* and *LmCys2* have been reported previously [8], [9] but sequence data are only in the form of BAC clones. The features of the proteins are listed below and in Table S4.

**Figure 2:**
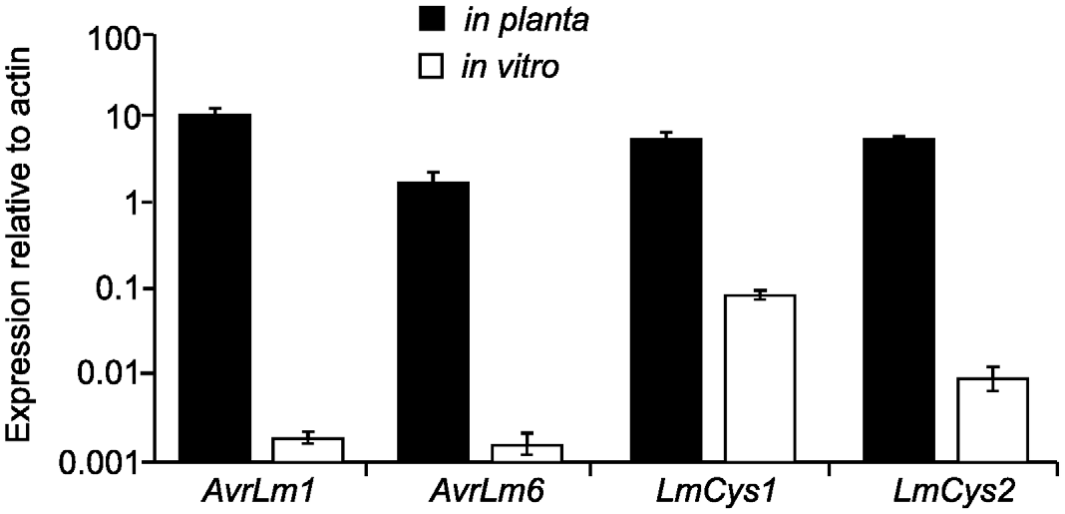
Quantitative reverse transcriptase (RT)-PCR analyses of *AvrLm6*, *AvrLm1, LmCys1* and *LmCys2*. At least 100 fold changes in expression were observed for each gene *in planta* compared to *in vitro* growth. RNA was prepared from seven day old cultures of isolates with the wild type alleles *AvrLm6-0*, *AvrLm1-0, LmCys1-0* and *LmCys2-0*, which were growing in 10% V8 juice. Additionally RNA was prepared from cotyledons of *B. napus* cv. Beacon 7 dpi with the same isolates. Two isolates were used. Transcript levels of each gene were compared to those of *L. maculans* actin within each sample. Data points are the average expression of each gene relative to actin determined from the two isolates, and three technical replicates for each sample. Expression relative to actin is presented as a log scale (Y-axis). Standard errors are represented.

The predicted LmCys1 protein (220 aa) contained eight cysteine residues and its single match was to a ‘secreted in xylem’ Six1 effector (also known as Avr3) of *Fusarium oxysporum* (26% identity, 40% similarity, accession number CAE55870.1) [26]. The predicted LmCys2 protein (247 aa), also containing eight cysteine residues, had no matches within the NCBI database. Both LmCys1 and LmCys2 were predicted to be secreted with signal peptides of 19 and 18 aa, respectively. To determine whether *LmCys1* and *LmCys2* were expressed highly *in planta*, which would be expected of genes encoding effector-like proteins, quantitative reverse transcriptase PCR (RT-PCR) analyses were performed. Transcript levels of *LmCys1* and *LmCys2 in planta* were six times higher than those of actin at seven days after inoculation of cotyledons of a susceptible *B. napus* cultivar (Figure 2). *LmCys1* and *LmCys2* were expressed at 0.1 and 0.01 times, respectively, that of actin in seven day *in vitro* cultures. Similar results were obtained with a second isolate (data not shown). This extremely high level of expression *in planta* compared to *in vitro* is similar to that seen for *AvrLm1* and *AvrLm6* (Figure 2) [8]. These characteristics (small cysteine-rich secreted proteins with no or few matches in databases and high *in planta* expression) strongly suggested that *LmCys1* and *LmCys2*, like *AvrLm1* and *AvrLm6*, encode effector-like proteins.

The LmTrans protein (373 aa) contained a DDE superfamily endonuclease domain predicted to be involved in efficient DNA transposition, and its best match was a putative transposase from *Stagonospora nodorum* (61% identity, 68% similarity, accession number CAD32687.1). The predicted LmGT protein (414 aa) was a putative glycosyltransferase with best matches to hypothetical protein PTRG_04076 of *Pyrenophora tritici-repentis* (89% identity, 95% similarity, EDU46914.1). The *LmMFS* protein (591 aa) belonged to the Major Facilitator Superfamily (MFS). This protein had best matches to putative protein SNOG_04897 from *S. nodorum* (74% identity, 82% similarity, EAT87288.2).

Since *LmCys1* and *LmCys2* had effector-like properties and thus putative roles in the plant-fungal interaction, these genes were sequenced in the 295 isolates. Five and two alleles were detected for *LmCys1* and *LmCys2*, respectively (Table 1), including the wild type alleles obtained from published BAC sequences. RIPCAL analysis showed that only one isolate, which was collected after the breakdown of sylvestris resistance had a RIP allele of *LmCys1*, and there were no deletion alleles, whereas there were no RIP alleles of *LmCys2*, but two isolates collected before breakdown of sylvestris resistance had a deletion of *LmCys2* (Table 1). Overall, the frequencies of individual alleles ranged from 99% for *LmCys2-0* to 0.3% for *AvrLm1-4*. The 295 isolates comprised 34 haplotypes based on alleles of *AvrLm1, AvrLm6*, *LmCys1* and *LmCys2* (Table S3).

Four non-coding, non-repetitive regions (Figure 1A) ranging in size between 247 and 657 bp were analysed, to see whether single copy non-coding regions, like the single copy genes, were affected by RIP mutation. These regions and the three other genes *LmTrans, LmGT* and *LmMFS* in this region (Figure 1A) were sequenced in a subset of 84 of the 295 isolates, which included isolates representing all 34 haplotypes described above (Table S3). Three, four, 14 and two alleles were detected for NC1, NC2, NC3 and NC4, respectively, whilst two, one, and three alleles were detected for *LmTrans*, *LmGT* and *LmMFS*, respectively (Table 4 and Table S5). The mutations giving rise to the alleles of NC1, NC2 and *LmMFS* were single non-RIP-like nucleotide substitutions (both synonymous and non-synonymous), and single base pair deletions. No polymorphisms were detected in *LmGT* and no RIP alleles were detected for NC1, NC2, *LmMFS* or *LmGT*. A single isolate had RIP alleles for NC4 and *LmTrans* in addition to NC3, *AvrLm6* and *LmCys1*. The NC3 region had an extremely high frequency of RIP mutation and the ten RIP alleles were all associated with *AvrLm6* RIP alleles. Based on all seven genes and four non-coding regions, 51 haplotypes were identified among the 84 isolates (Table S6).

**Table 4:** Nucleotide changes in non-coding, non-repetitive regions within the *AvrLm1-LmCys2* genomic region in Australian isolates of *Leptosphaeria maculans*.

Regiona, b	Allele	Number of isolates (frequency %)	Nucleotide changes				RIP dominance score
			No.	Type			
				CpA to TpA	TpG to TpA	Other	
NC1	0	64 (76.2)	0	0	0	0	0
	1	1 (1.2)	1	0	0	1 ( C to G)	0
	2	19 (22.6)	1	0	0	1 (T to C)	0
NC2	0	53 (63.1)	0	0	0	0	0
	1	27 (32.1)	1	0	0	1 ( T to A)	0
	2	1 (1.2)	1	0	0	1 (A to T)	0
	3	3 (3.6)	3	0	0	1 (T to A, del G, del T)	0
NC3	0	67 (79.6)	0	0	0	0	0
	1	1 (1.2)	1	0	0	1 (T to C)	0
	2	1 (1.2)	1	0	0	1 (A to G)	0
	3	1 (1.2)	1	0	0	1 (C to G)	0
	4	2 (2.4)	28	9	7	12 (all G to A or C to T)	1.5
	5	1 (1.2)	29	9	7	13 (all G to A or C to T)	1.5
	6	1 (1.2)	29	9	7	13 (all G to A or C to T)	1.3
	7	1 (1.2)	30	9	7	14 (all G to A or C to T)	1.5
	8	1 (1.2)	38	13	12	13 (all G to A or C to T)	3.6
	9	1 (1.2)	34	9	11	14 (all G to A or C to T)	2.0
	10	1 (1.2)	38	13	11	14 (all G to A or C to T)	3.0
	11	2 (2.4)	39	9	12	18 (all G to A or C to T)	2.1
	12	3 (3.6)	40	9	12	19 (all G to A or C to T)	2.1
	13	1 (1.2)	29	8	8	12 (all G to A or C to T)	1.6
NC4	0	83 (98.8)	0	0	0	0	
	1	1 (1.2)	9	4	0	5 (all G to A or C to T)	4.0

These isolates represented all 34 haplotypes based on *AvrLm1*, *AvrLm6*, *LmCys1* and *LmCys2* alleles.

a The reference sequences are CT485790 (designated as NC1-0), CT485667 (NC2-0), CT485649 (NC3-0) and CT485669 (NC4-0) identified from isolate v23.1.3 [8], [9].

b The sizes of the amplified products were 517 bp for NC1-0, 496 bp for NC2-0, 657 bp for NC3-0 and 139 bp for NC4-0.

### Distribution and extent of RIP mutations

**Figure 3:**
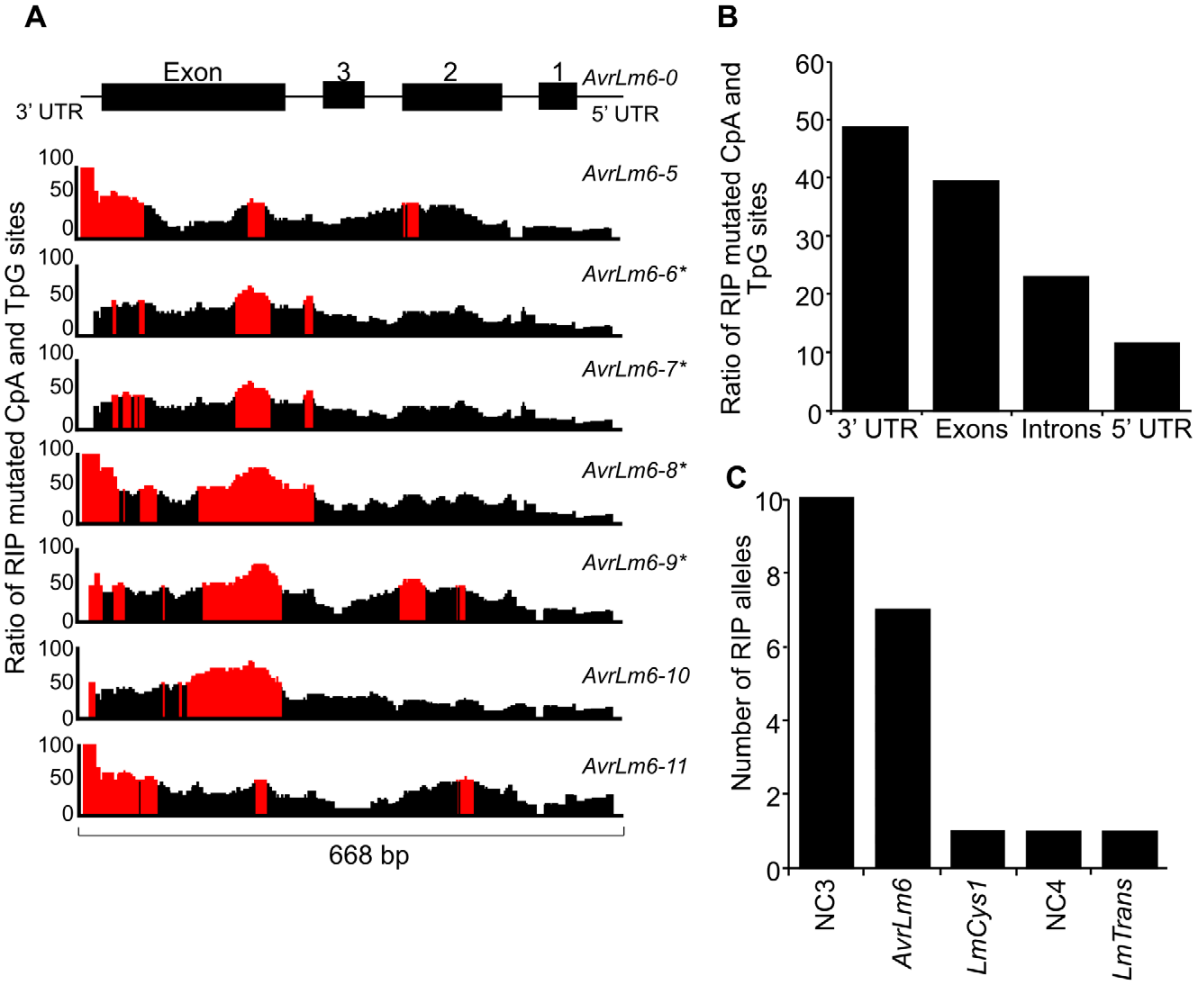
Distribution of RIP mutations across the *AvrLm1-LmCys2* genomic region in *Leptosphaeria maculans*. (A) The ratio of mutated CpA or TpG sites within the seven *AvrLm6* RIP alleles compared to the number of available CpA and TpG nucleotides present in the wild type *AvrLm6-0* allele over a 100 bp window. Regions where greater than 50% of potential RIP sites are mutated are highlighted in red. There is a higher proportion of RIP mutations towards the 5′ end of the region (3′ end of the *AvrLm6* gene). *AvrLm6-0, -1, -2, -3* and *-4* alleles do not display RIP mutations. (B) The average ratio of RIP mutations within the untranslated regions (UTRs), exons and introns for the seven RIP alleles of *AvrLm6* relative to the number of potential RIP sites in the wild type sequence. The 3′ UTR and exons are undergoing the highest frequency of RIP. (C) The number of RIP alleles for each of the genes and non-coding, non-repetitive (NC) regions analysed across the *AvrLm1-LmCys2* genomic region in 84 isolates (see also Figure S2). The number of RIP alleles is highest for NC3 and decreases in the genes and non coding regions downstream, consistent with RIP occurring in a directional manner.* represent RIP alleles of *AvrLm6* that are not transcribed and that confer a virulence phenotype on an *Rlm6*-containing cultivar. The remaining RIP alleles of *AvrLm6* were not tested for virulence towards *Rlm6*.

The distribution and degree of RIP mutation across each allele of each gene was determined. This was represented as a ratio of the number of mutated CpA and TpG sites relative to number of potential RIP sites in isolate v23.1.3 in a 100 bp rolling window. The seven RIP alleles of *AvrLm6* had the highest frequency of RIP towards the 3′ end (Figure 3A). The frequency of RIP was higher within the 3′ UTR and the exons, than in the introns and 5′ UTR (Figure 3B). The ten RIP alleles of NC3 and single RIP allele of *LmCys1* showed the highest frequency of RIP at their 5′ ends, whilst RIP mutations were evenly distributed throughout the single RIP allele of *LmTrans* (Figure S2), the latter gene being the furthest 3′ characterised single copy sequence affected by RIP mutation within the *AvrLm1*-*LmCys2* genomic region (Figure 3C). The NC3-*Avrlm6* and *LmCys1*-NC4-*LmTrans* single copy regions in which RIP mutations were detected, were separated by 37 kb of repetitive elements (Figure 1B). Both these single copy regions were closer to upstream repetitive elements (<342 bp) than to downstream repetitive elements (>1 kb) (Figure 1B). The degree of RIP within these single copy regions was proportional to their proximity to repetitive elements, and thus a gradient of RIP mutations was apparent in a 5′ to 3′ direction (Figures 1B and 3B).

Since amongst these single copy regions the degree of RIP mutations was highest in NC3, a repeat region (620 bp) directly upstream was analysed to see if it was severely RIP-affected and thus might act as a point of ‘leakage’ of RIP into NC3 and *AvrLm6* (Figure 1B). BLAST analysis of the genomic sequence of isolate v23.1.3 revealed 293 copies of this repeat. RIPCAL analysis showed that the repeat directly upstream of NC3 was amongst the most highly RIP-affected copy of that repeat in the genome.

### Transcription of *AvrLm1*, *AvrLm6*, *LmCys1* and *LmCys2* alleles

The mutations of *AvrLm1*, *AvrLm6*, *LmCys1* and *LmCys2* (deletions or RIP mutations including stop codons) would be expected to lead to lack of transcription of these alleles. This hypothesis was assessed in a range of isolates seven days after inoculation of cotyledons of *B. napus* cv. Beacon, which all the isolates could attack. Primers designed to amplify 500–700 bp products within the coding region of these genes were used in end-point RT-PCR (Table 5). Isolates with either *AvrLm1-0* or *AvrLm1-1* allele had an *AvrLm1* transcript of the appropriate size. As expected, isolates with the deletion allele had no transcript. The *AvrLm6-0*, *AvrLm6-1* or *AvrLm6-2* alleles were expressed, whilst the RIP alleles, *AvrLm6-6*, *AvrLm6-7*, *AvrLm6-8* or *AvrLm6-9* were not. As expected, no expression of *AvrLm6* or of *LmCys2* was detected in isolates with the deletion alleles of these genes. Isolates with *LmCys1-0*, *LmCys1-1* or *LmCys1-2* alleles had an *LmCys1* transcript, whilst the isolate with the RIP allele, *LmCys1-4*, did not. Actin transcripts were detected in all isolates.

**Table 5:** Expression analysis of *AvrLm1*, *AvrLm6*, *LmCys1* and *LmCys2* alleles in *Leptosphaeria maculans* isolates *in planta*.

Isolate	Genotype (allele)				Gene expression (7 days post inoculation)				
	*AvrLm1*	*AvrLm6*	*LmCys1*	*LmCys2*	*AvrLm1*	*AvrLm6*	*LmCys1*	*LmCys2*	actin
05P031	*0*	*1*	*0*	*0*	+	+	+	+	+
06P042	*0*	*9* a	*1*	*0*	+	−	+	+	+
04P012	*1*	*0*	*1*	*0*	+	+	+	+	+
06P040	*1*	*del*	*1*	*0*	+	−	+	+	+
IBCN18	*1*	*del*	*2*	*del*	+	−	+	−	+
05P033	*del*	*2*	*1*	*0*	−	+	+	+	+
05P034	*del*	*2*	*1*	*0*	−	+	+	+	+
06S013	*del*	*6* a	*1*	*0*	−	−	+	+	+
06S014	*del*	*7* a	*1*	*0*	−	−	+	+	+
06S039	*del*	*8* a	*4* a	*0*	−	−	−	+	+

Cotyledons of 14 day old seedlings of *Brassica napus* cv. Beacon were infected with conidia of individual *L. maculans* isolates. Seven days post inoculation, tissue was harvested and RNA extracted from 20 inoculation sites per isolate. Expression of genes was determined using end point RT-PCR. ‘+’ band of expected size. ‘−’ no band.

a alleles associated with RIP.

### Rate of mutations of genes

To determine the rates of mutations, genes were analysed by phylogeny-based likelihood ratio tests (LRT) implemented in the program HyPhy [27]. These tests suggested that nucleotides of *AvrLm1* and *LmMFS* mutated at a constant rate, i.e. mutations did not deviate significantly (p = 0.544) from a clock-like rate of evolution. In contrast, an accelerated mutation rate was detected for *AvrLm6* and *LmCys1* loci (P<0.001) compared to expectations under constant (i.e. clock-like) evolution. However, when RIP alleles were excluded, mutations evolved at a clock-like rate (Table 6). Genetic divergence between haplotypes was calculated using Molecular Evolutionary Genetics Analysis (MEGA) software [28]. Relative rates of sequence evolution of non-RIP alleles were much lower than those of RIP alleles (Table 6). To determine whether the seven proteins were undergoing positive selection, the rates of non-synonymous and synonymous substitutions were compared. All RIP alleles were excluded from this analysis since they contained multiple stop codons. Evolution of codon changes within AvrLm1 and LmCys1 was best explained by a model of positive selection, as shown by a likelihood ratio test implemented using two complementary approaches, the sitewise likelihood-ratio (SLR) and phylogenetic analysis by maximum likelihood (PAML) methods (Table 7). This interpretation was supported by the finding of positive selection at a single codon site in AvrLm1 and at three sites in LmCys1 (Table 7). Analysis of the AvrLm6 protein using the SLR approach suggested a single amino acid (codon 54) may be undergoing positive selection; however, this finding was not supported by the PAML analysis. Similar analyses could not be performed on LmCys2, LmGT or LmTrans as these genes had fewer than three alleles.

**Table 6:** Analysis for the presence of a molecular clock, and relative rates of sequence evolution for genes within the *AvrLm1-LmCys2* genomic region of *Leptosphaeria maculans*.

Gene	Analysis of molecular clocks (HyPhy) a				Relative rates of substitution (MEGA) b	
	LogLh-no clock	LogLh-clock	p-value	Interpretation	Nucleotide (K2P)	Interpretation
*AvrLm1*	−1073.67	−1077.13	0.544	Clock-like	0.002 (0.001)	
*AvrLm6*	−1578.58	−1633.62	<0.001	Accelerated	0.051 (0.006)	
excl. RIPs	−956.75	−963.27	0.111	Clock-like	0.003 (0.001)	Genetic distances <10 fold lower with RIP alleles excluded
*LmCys1*	−1175.04	−1240.44	<0.001	Accelerated	0.046 (0.006)	
excl. RIPs	−964.78	−968.33	0.311	Clock-like	0.005 (0.002)	Genetic distances <10 fold lower with RIP alleles excluded
*LmTrans*	*NC*	*NC*	*NC*	*NC*	0.086 (0.008)	
excl. RIPs	*NC*	*NC*	*NC*	*NC*	*NC*	
*LmGT*	*NC*	*NC*	*NC*	*NC*	*NC*	
*LmMFS*	−2726.22	−2729.52	0.159	Clock-like	0.001 (0.001)	
*LmCys2*	*NC*	*NC*	*NC*	*NC*	*NC*	

Presence of a molecular clock was analysed using a phylogeny-based likelihood ratio test.

MEGA was used to infer relative rates of sequence evolution by calculating means of genetic distances (Kimura-2-Parameter (K2P) between haplotypes.

a When RIP alleles were excluded from HyPhy analysis (excl. RIPs), p values for all genes became non-significant (clock-like).

b Since both HyPhy and MEGA approaches are phylogeny-based, a minimum of three distinct alleles are required for analysis. Genes with less than three alleles could not be analysed (*NC*).

**Table 7:** Analysis of positive selection on amino acids of proteins encoded within the *AvrLm1-LmCys2* genomic region of *Leptosphaeria maculans*.

Protein	SLR approach		PAML approach (M7 vs. M8)			
	Positively selected codons	*P* value	LRT statistics a	*P* value	Positively selected codons	*P* value
AvrLm1	125	0.002	8.126	0.017	125	0.001
AvrLm6	54	0.003	0.214	0.900	54	NS
LmCys1	53	<0.001	24.018	<0.001	53	0.002
	121	<0.001			121	<0.001
	134	0.002			134	<0.001
LmTrans	*NC*	*–*	*NC*	*–*	*NC*	*–*
LmGT	*NC*	*–*	*NC*	*–*	*NC*	*–*
LmMFS	none	–	0.00	1.00	none	–
LmCys2	*NC*	*–*	*NC*	*–*	*NC*	*–*

Evidence for non-neutral selection was assessed by comparing the rate of non-synonymous and synonymous substitutions using two approaches, SLR and PAML [52], [53]. These approaches are phylogeny-based and require a minimum of three distinct alleles for analysis, so values for LmTrans, LmGT and LmCys2 were not calculated (*NC*). For the PAML approach, the comparison included the likelihood estimates of the neutral null model (M7) and the alternative model of positive selection (M8). RIP alleles were excluded from these analyses since such alleles encode sequences with stop codons.

NS  =  not significant.

a LRT statistics are compared against a χ^2^ distribution with two degrees of freedom.

### Evolution and phylogenetic relationships of RIP and deletion alleles

**Figure 4:**
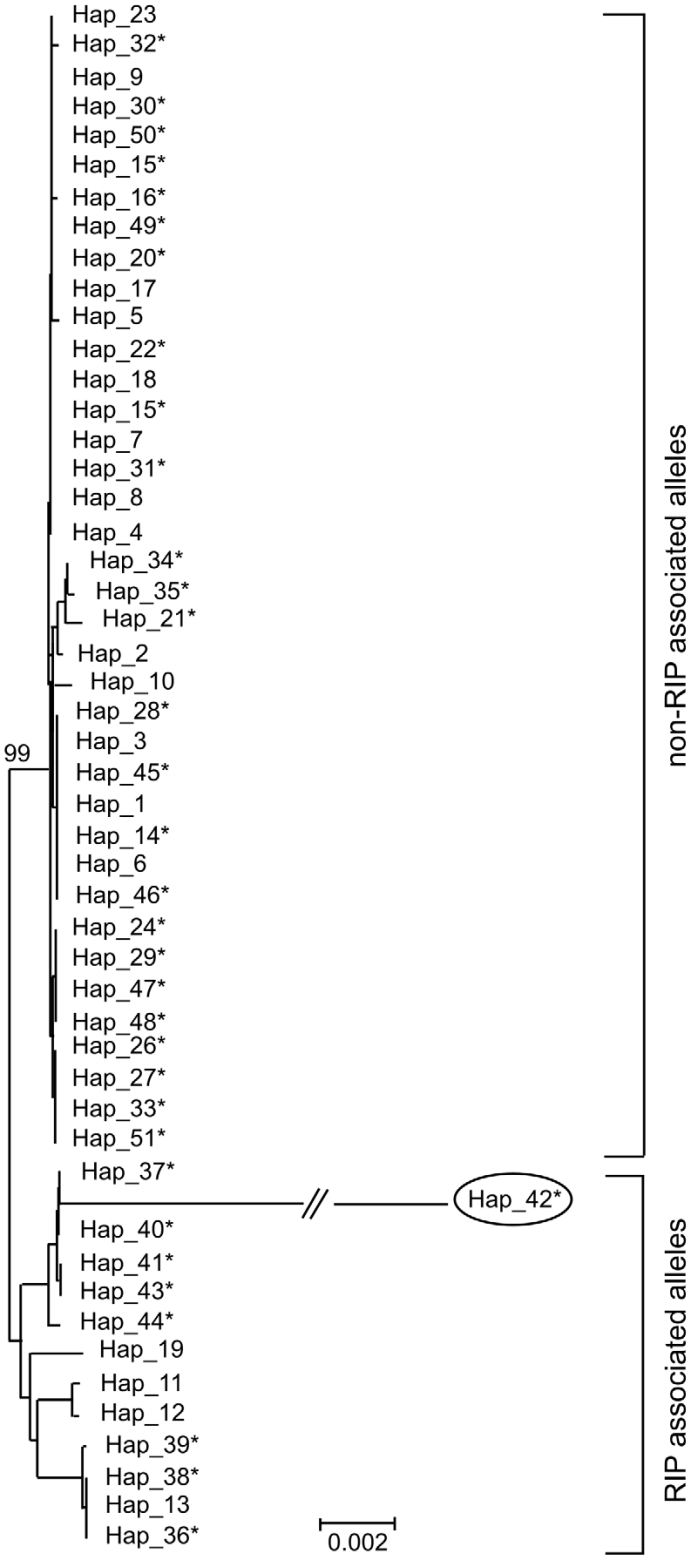
Genetic diversity and phylogenetic relationships between haplotypes of *Leptosphaeria maculans.* Un-rooted strict consensus of 1000 ML trees (-ln L  =  16381.75) constructed from the concatenated DNA sequences of the seven genes and four non-coding, non-repetitive regions of the *AvrLm1-LmCys2* genomic region. All CpA to TpA and TpG to TpA nucleotide changes were removed from the data set prior to analysis. Haplotypes (see Table S6) associated with RIP alleles cluster in a distinct clade with high bootstrap support (99%), suggesting a single origin. In contrast, haplotypes related to deletion alleles (*) are associated with multiple clades suggesting multiple origins. Note that RIP associated alleles have much longer branches (i.e. larger genetic distance) due to an accelerated evolution compared to non-RIP alleles (see Table 6). Haplotype 42 (circled) has RIP alleles at five of the loci examined (NC3, *AvrLm6*, *LmCys1*, NC4 and *LmTrans*), resulting in an exceptionally long branch.

Two hypotheses for the evolution of RIP alleles, monophyly (having a single origin or having evolved only once) and polyphyly (multiple origins or having evolved several times independently) were tested by comparing tree topologies using the Kishino–Hasegawa (KH) test [29]. For both the Maximum Parsimony (MP) and Maximum Likelihood (ML) approach, trees based on the assumption of a monophyletic origin of RIP alleles performed significantly better than those based on polyphyletic origin (Table 8). The phylogenetic relationship of all detected haplotypes is depicted in Figure 4. The RIP and non-RIP associated alleles form two distinct clusters, supporting the hypothesis of a single origin of RIP alleles. In contrast, haplotypes associated with gene deletions are associated with multiple clades of the tree and have probably arisen several times.

**Table 8:** Hypothesis-testing of multiple (polyphyletic) vs. single (monophyletic) origin of RIP mutation-associated haplotypes of *Leptosphaeria maculans*.

Tree	Tree score a	Score difference^b^	*P*
Maximum parsimony (MP) (gaps treated as missing)			
Monophyletic origin of RIP	15488	10	<0.01
Polyphyletic origin of RIP	15478	best	
Maximum parsimony (MP) (gaps treated as fifth state)			
Monophyletic origin of RIP	15550	32	<0.01
Polyphyletic origin of RIP	15518	best	
Maximum likelihood (ML)			
Monophyletic origin of RIP	15345	58	<0.01
Polyphyletic origin of RIP	15287	best	

Kishino–Hasegawa tests were used to assess different hypotheses of RIP evolution by comparing tree topologies as implemented in PAUP*. The probabilities (*P*) of obtaining better trees were assessed using two-tailed tests, the full optimization criterion and 1000 bootstrap replicates.

a Tree scores refer to branch lengths for the tree topologies. For the maximum likelihood analysis, tree scores are given as –ln of branch length.

b ‘Best’ refers to the ‘shortest’ tree, which is likely to be the most accurate.

## Discussion

### Selection pressure on frequencies of *AvrLm1* and *AvrLm6* alleles is imposed by extensive sowing of cultivars with *Rlm1* resistance

Breakdown of disease resistance has been observed in other plant fungal pathogen systems where fungal populations evolve rapidly (for review see [30]). In the canola- *L. maculans* interaction described here, strong selection pressure was exerted on the *AvrLm1* locus, due to extensive sowing of sylvestris cultivars with *Rlm1*, which was consistent with the finding of a rapid increase in the frequency of isolates with virulent (*avrLm1*) alleles after the breakdown of resistance. Surprisingly the frequency of isolates virulent (with the *avrLm6* allele) towards *Rlm6* increased, although cultivars with polygenic or with sylvestris resistance have not been shown to contain *Rlm6* [17], [31]. The linkage and genomic location of *AvrLm1* and *AvrLm6* may have led to a selective sweep whereby selection at *AvrLm1* affected the frequency of *avrLm6* alleles through ‘hitchhiking’. Thus strong selection imposed by wide-spread deployment of a plant resistance gene that favors a complementary effector allele in a pathogen could affect evolution of closely-linked effector genes. It is intriguing that in France recurrent sowing of *Rlm6*-containing cultivars in localised field trials led to an increase in *avrLm6* isolates and a corresponding increase in isolates avirulent at the *AvrLm1* locus [19]. This difference may be due to the different targets of selection pressure - *Rlm6* in France and *Rlm1* in our study.

This situation whereby selection pressure on one gene affected allele frequencies of another may be partly due to the presence of these two effector genes in a repeat-rich region, where there is a low recombination frequency. Effectors in other fungi are present in repeat-rich regions. In the rice blast fungus, *Magnaporthe oryzae*, avirulence gene *Avr-Pita* is located 48 bp from telomere repeats whilst *Avr1-CO39* is associated with transposable elements [32], [33]. An avirulence gene (SIX1) of *Fusarium oxysporum* f.sp. *lycopersici* is flanked by transposable elements [34] and other effectors are localised on a single, transposon-rich chromosome [35]. Toxin-encoding genes, *Tox3 and ToxA*, are located next to repetitive elements in *Stagonospora nodorum* [36], [37], [38] and effectors are in repeat-rich regions of the genome of the oomycete, *Phytophthora infestans* [39]. In the sequenced isolate of *L. maculans, AvrLm1* and *AvrLm6* are located amongst multiple copies of long terminal repeat retrotransposons, namely *Pholy*, *Olly*, *Polly* and *Rolly*, which are generally incomplete. Furthermore the distribution and number of these elements within this genomic location varies considerably between isolates [20], [22]. The presence of effector genes within such regions is suggested to promote their adaptation and diversification when exposed to strong selection pressure [40]. Rep and Kistler have speculated that the presence of highly repetitive regions containing transposons, may promote mutation of resident effector genes [41].

### Repeat-induced point mutations may be ‘leaking’ from adjacent inactivated transposable elements into single copy regions

The genes and non-coding regions undergoing RIP within the *AvrLm1- LmCys2* gene region are single copy and therefore are not expected to be targeted by RIP. Two explanations for the presence of RIP mutations in these genes are as follows. Firstly, if these genes are the result of an ancestral duplication, RIP mutation may be acting directly on them. However, Fudal et al showed that no closely related paralogs of *AvrLm6* exist in the genome of isolate v23.1.3 suggesting that RIP would not be targeting these sequences due to a duplication event [22]. Alternatively, the *AvrLm6* locus may be completely or partially duplicated in isolates where RIP was detected. However, southern hybridisations suggest only a single copy of the *Avrlm6* locus in isolates where RIP was detected, which does not support the possibility of RIP being targeted to this locus. A more likely explanation is that RIP mutations ‘leak’ from adjacent repetitive sequences. As RIP mutation is traditionally observed to be restricted to repetitive regions and not single copy regions, leakage of RIP mutation might occur within a relatively short distance of a RIP-affected repeat, as suggested by Fudal et al [22]. Indeed, this has been reported in *N. crassa* whereby leakage of RIP was detected in single copy sequences at least 930 bp from the boundary of neighbouring duplicated sequences [42]. This is consistent with our finding of a high frequency of RIP mutations in single copy regions of *L. maculans* with the degree of RIP mutation being proportional to the proximity of flanking repetitive elements. The potential ‘leakage’ of RIP mutations into closely linked effector genes highlights the power of this process to lead to major evolutionary changes to genes such as effectors that play an important role in the lifestyle of an organism.

### Deletion alleles of *AvrLm1* and *AvrLm6* had multiple origins whilst RIP alleles had a single origin

All haplotypes associated with RIP alleles of *AvrLm6* and *LmCys1* appeared to have a single origin indicating that the event that led to the RIP occurred only once. Furthermore, haplotypes associated with the virulent *AvrLm6-2* allele (cysteine substitution) arose as a single clade. These single origins suggest that events leading to both the RIP mutations and non-RIP amino acid substitutions are much rarer than those leading to the deletion mutations. The RIP event occurred after the breakdown of sylvestris resistance, as isolates with RIP mutations were not detected before this time. This rapid appearance of RIP mutations is not unprecedented; such mutations have been shown to occur after one generation in *L. maculans*. When transformants with several tandemly linked copies of a hygromycin resistance gene were crossed with a wild type strain, none of the progeny were resistant to hygromycin due to the presence of multiple stop codons generated by RIP mutations in the hygromycin resistance gene [43].

Mutations associated with resistance to azole fungicides in *Mycosphaerella graminicola* also are derived from a single origin. Resistance emerged only once following strong selection due to widespread use of azole fungicides [44]. In both the *M. graminicola* and *L. maculans* examples, a similar phylogeny was found despite differences in origin and type of evolutionary pressure. In *L.maculans* haplotypes associated with deletion alleles conferring virulence towards *AvrLm1* and *AvrLm6* appeared to have a polyphyletic origin. Isolates with these deletions were detected prior to 2004 when the resistance breakdown occurred, albeit at a much lower frequency than afterwards. Haplotypes associated with deletion of both *AvrLm1* and *AvrLm6* might have been derived directly from the ancestral wild type rather than via the deletion of one gene followed by that of the second.

### Deletions, non-synonymous and RIP mutations can confer virulence

Deletions, RIP mutations and non-RIP amino acid substitutions conferred virulence at the *AvrLm6* locus, whilst only deletions were responsible for virulence at the *AvrLm1* locus. Similar types of mutations were detected in French populations of *L. maculans* isolates [22]. The finding of a virulence allele of *AvrLm6* arising from a non-synonymous, non-RIP like mutation, of a glycine to cysteine substitution, was intriguing. In other avirulence proteins the loss rather than the gain of cysteine through non-synonymous substitutions confers virulence. For example, in the AVR4 protein of *Cladosporum fulvum*, loss of cysteine residues renders the isolate virulent towards tomato cultivars with the corresponding *Cf-4* resistance gene [45]. The *AvrLm6-2* allele of *L. maculans* gives rise to a protein with seven rather than six cysteine residues in the protein encoded by *AvrLm6-0*. This latter protein is proposed to have two disulphide bridges between C^109^ and C^130^, and C^103^ and C^122^ [8] on the basis of the SCRATCH disulphide bond prediction program [46]. The program predicts that the presence of the additional cysteine (C^123^) in the *AvrLm6-2* allele would result in a third disulphide bridge, between C^26^ and C^123^.

As well as the mechanisms leading to inactivation of alleles described above, some of the proteins were undergoing non-RIP amino acid substitutions which did not lead to a change in phenotype. Some of these mutations, in AvrLm1 and LmCys1, were the results of positive selection, which favours new mutations that confer a fitness advantage and thus lead to an increase in gene diversity [12], [47]. Positive selection has been detected in pathogen effector genes including the avirulence gene that encodes NIP in *Rhynchosporium secalis* [47] and in genes encoding host-specific toxins such as *S. nodorum* ToxA [36], [48]. In contrast to *AvrLm1, AvrLm6* and *LmCys1*, the remaining genes in the 520 kb AT- rich region, including *LmCys2* showed very little variation. Despite positive selection driving amino acid substitutions within some of the effector-like proteins, deletion and RIP mutations are by far the major mechanisms leading to virulence at the *AvrLm1* and *AvrLm6* loci.

## Materials and Methods

### *Brassica* cultivars and *Leptosphaeria maculans* isolates

Stubble of cultivars of *B. napus* and *B. juncea* infested with *L. maculans* was collected each year from 1997 to 2008 from 25 locations across Australia (Table S1). For instance, stubble from a crop sown in 2003 was collected from the field in 2004 and isolates were then cultured from it. Cultivars with ‘polygenic’ resistance (Beacon, Dunkeld, Emblem, Grace, Hyden, Jade, Pinnacle, Skipton, Pinnacle and Tornado TT) had one or more *Rlm* genes, but none had *Rlm1* nor *Rlm6*. The identity of resistance genes in some of these cultivars has been reported [44]. The category of ‘sylvestris resistance’ refers to cultivars (Surpass 400, Surpass 501TT, Surpass 603CL, 45Y77 and 46Y78) with resistance derived from *B. rapa* spp. *sylvestris* [14] have *Rlm1* and *RlmS* [17]. The category of ‘juncea’ resistance refers to cultivars and lines of *B. juncea* (cv. Dune and lines JC05002, JC05006 and JC05007). Stubble of the latter two categories was not collected prior to 2004. From 2004 onwards although cultivars with sylvestris resistance were withdrawn from sale, these lines were grown in yield trials across Australia, and stubble was collected from them. Isolates (287) were cultured from individual ascospores discharged from stubble collected the previous year as described previously [15]. In addition, eight Australian isolates collected in 1987 and 1988 were analysed. All isolates were maintained on 10% Campbell's V8 juice agar.

### Virulence testing

Virulence of a subset of isolates was tested on three *B. napus* and one *B. juncea* cultivars. The *B. napus* cv. Beacon and cv. Q2 are susceptible controls that all isolates could attack. Cultivar Columbus contains *Rlm1* and *Rlm3* and *B. juncea* cv. Aurea contains *Rlm5* and *Rlm6* [3]. No *Rlm1*-only or *Rlm6*-only cultivars were available. Cotyledons of 14-day old seedlings were wounded and inoculated with conidia of individual isolates representing different haplotypes for *AvrLm1* and *AvrLm6*. Symptoms were assessed at 10, 14 and 17 days post-inoculation (dpi) and pathogenicity scores determined at 17 dpi by scoring lesions on a scale from 0 (no darkening around wounds) to 9 (large grey-green lesions with profuse sporulation). Mean pathogenicity scores (determined from 40 inoculation sites) ≤3.9 were assigned as an avirulent phenotype whilst scores ≥4.0 were assigned as a virulent phenotype [17].

### Gene identification and primer design

Non-coding, non-repetitive regions in the *AvrLm1-LmCys2* genomic region and genes 3′ of *AvrLm6* (Figure 1) were identified using published information [22] and by BLAST searches. Primers were designed upstream and downstream of start and stop codons to allow analysis of the sequences of entire open reading frames. For transcriptional analyses, primers were designed to amplify a 500–700 bp region of the coding sequence, flanking an intron where possible. All primers were designed using the program Primer3 [49] (Table S7). Primers to amplify the mating type locus and actin have been described previously [8], [24].

The sequence information for all genes has been deposited in GenBank with the following accession numbers; *AvrLm1*, AM084345 [1], *AvrLm6*, AM259336 [2], *LmCys1*, GU332625, *LmTrans*, GU332626, *LmGT*, GU332627, *LmMFS*, GU332628 and *LmCys2*, GU332629.

### Allele genotyping

Genomic DNA was isolated from mycelia. Conditions for all PCR experiments were 95°C for 3 min; 35 cycles of 95°C for 30 sec, 59°C for 30 sec and 72°C for 1 min; 72°C for 6 min. PCR products were purified using QIAquick PCR purification kit (Qiagen) and sequenced using BigDyeTM terminator cycling conditions. Sequences were analysed using Sequencher v 4.0.5. Deletion genotypes were assigned if no band was produced following amplification with the *AvrLm1*, *AvrLm6* or *LmCys2*-specific primers. Amplification of the mating type locus was a positive control for DNA quality. In a subset of eight isolates, deletion alleles were confirmed by Southern analysis of genomic DNA that had been digested with *Hind*III and hybridised with the appropriate probe (Figure S1). Additionally, PCR screens were performed on genomic DNA from two (for *LmCys2*) to 25 (for *AvrLm6*) isolates using multiple primer sets that amplify specific regions of the *AvrLm1*, *AvrLm6* and *LmCys2* gene regions. These amplifications confirmed that the entire locus was deleted in all isolates tested (data not shown).

Allele sequences were analysed by RIPCAL for the presence of RIP mutations [25]. RIPCAL generates a RIP dominance score, which is the frequency of the dominant dinucleotide RIP mutation (in this case CpA→TpA) relative to the sum of the alternative mutations (CpC→TpC, CpG→TpG and CpT→TpT). Sequences with RIP dominance scores >1 are considered to be highly RIP-affected. The ‘model’ sequence used for all RIPCAL analyses was the ‘wild type’ allele (designated with a *-0* suffix) from the isolate (v23.1.3) whose genome has been sequenced. The spatial distribution of RIP was assessed for each gene and four non-coding regions by comparing the ratio of mutated CpA or TpG sites detected by RIPCAL, relative to the number of available CpA and TpG nucleotides present within the wild type allele over a 100 bp rolling window.

### Expression analysis

Ten infected cotyledons of *B. napus* cv. Beacon were harvested at 7 dpi. Necrotic tissue surrounding the inoculation wounds of each cotyledon was harvested using a hole punch (diameter 8 mm). Total RNA was purified from this tissue using the RNeasy Plant Mini Kit (Qiagen) and was treated with DNaseI (Invitrogen) before cDNA was synthesized using a first strand cDNA synthesis kit and an oligo-dT primer. End point RT-PCR was used to assess expression of *AvrLm1*, *AvrLm6*, *LmCys1*, *LmCys2* and actin.

Quantitative RT-PCR was used to determine levels of expression of *AvrLm1*, *AvrLm6, LmCys1* and *LmCys2 in planta* and *in vitro* culture. RNA was prepared from seven day old cultures of isolates with the wild type alleles of these genes, which were growing in 10% V8 juice. Additionally RNA was prepared from cotyledons of *B. napus* cv. Beacon 7 dpi infected with isolates with the wild type alleles of these genes. Total RNA and cDNA synthesis was performed as described above. Controls lacking reverse transcriptase were included. Quantitative RT-PCR was performed using Rotor-Gene 3000 equipment (Corbett Research, Australia) and QuantiTect SYBR Green PCR kit (QIAgen). A standard curve of amplification efficiency of each gene was generated from purified RT-PCR products [50]. Diluted RT product (1 μl) was added to 19 μl of PCR mix and subjected to 40 cycles of PCR (30 s at 94°C, then 60°C and then 72°C). All samples were analysed in triplicate. The amplified product was detected every cycle at the end of the 72°C step. Melt curve analysis after the cycling confirmed the absence of non-specific products in the reaction. The fluorescence threshold (Ct) values were determined for standards and samples using the Rotor-Gene 5 software. Ct values were exported to Microsoft Excel and analysed [51]. Actin was used as a reference gene.

### Phylogenetic analyses

Deviation from a constant rate of molecular evolution within the data sets (i.e. a “molecular clock”) was assessed using the phylogeny-based likelihood ratio test (LRT) implemented in the program HyPhy [27]. To estimate the contribution of the RIP alleles, likelihoods were calculated both for the total data sets and for data sets excluding RIP alleles. MEGA was also used to infer relative rates of sequence evolution by calculating means of genetic distances (Kimura-2-Parameter) between haplotypes.

Evidence for non-neutral evolution was assessed using two complementary approaches by comparing the rate of non-synonymous substitutions with the rate of synonymous substitutions (dN/dS  =  ω). Firstly, the analysis was based on the “sitewise likelihood-ratio” method as implemented in the SLR software package [52]. The test consists of performing a likelihood-ratio test on a site-wise basis, testing the null model (neutrality, ω = 1) against an alternative model ω≠1 (i.e. purifying selection ω<1; positive selection ω>1). Secondly, dN/dS  =  ω was tested using a phylogenetic analysis based on maximum likelihood as implemented in the PAML software package [53]. Two codon substitution models were compared via likelihood ratio tests (LRT). The comparison included the likelihood estimates of the neutral null model (M7) and the alternative model of positive selection (M8). RIP alleles were excluded from these analyses since such alleles encode sequences with stop codons.

To test different hypotheses of emergence of haplotypes associated with RIP alleles (Table S6), tree topologies using concatenated DNA sequences of all the genes (*AvrLm1, AvrLm6, LmCys1, LmTrans, LmGT, LmMFS* and *LmCys2*) and non-coding, non-repetitive regions (NC1-4) were generated and compared using the Kishino–Hasegawa (KH) test [29] as implemented in PAUP* 4.0b 10. Since the RIP mechanism produces the same mutations at specific sites, it is likely that formerly unrelated nucleotide sequences converge, leading to the false impression of similarity due to common descent. To avoid this bias, all CpA to TpA and TpG to TpA nucleotide changes were removed from the data set prior to inferring the phylogenetic relationships of haplotypes. Two alternative hypotheses were then compared; (i) haplotypes containing RIP alleles were monophyletic, i.e. they emerged only once. Trees representing this hypothesis were “constrained” by restricting RIP alleles to cluster only amongst each other (ii) haplotypes containing RIP alleles were polyphyletic, i.e. they emerged several times independently. Trees representing this alternative hypothesis were “unconstrained”, i.e. the pairing of particular alleles in the topology was not restricted. One thousand trees were generated representing each hypothesis, and the probabilities (*P*) of obtaining better trees were assessed using two-tailed tests, the full optimization criterion and 1000 bootstrap replicates. The KH test was conducted for trees constructed under the maximum likelihood and the maximum parsimony criterion.

The phylogenetic relationship among isolates based on the concatenated DNA sequences of all genes and non-coding, non-repetitive regions was assessed by PAUP* using maximum likelihood. Tree searches were conducted with the “fast-stepwise-addition” option and 1000 bootstrap replicates to assess statistical significance of nodes. The GTR-model with estimated substitution-rate matrix was used to evaluate molecular rate constancy.

## Supporting Information

Figure S1 Confirmation of deletion alleles for *AvrLm1, AvrLm6* and *LmCys2* by Southern analysis of genomic DNA. (A) Hybridisation of an *AvrLm1* probe to genomic DNA of two isolates that produced an amplicon after PCR using primers specific for *AvrLm1* (lanes 1 and 7) and five isolates that did not (lanes 2–6). (B) Hybridisation of an *AvrLm6* probe to genomic DNA of five isolates that produced an amplicon after PCR using primers specific for *AvrLm6* (lane 1–3 and 7–8) and three isolates that did not (lanes 4–6). (C) Hybridisation of a *LmCys2* probe to genomic DNA of a single isolate that produced an amplicon after PCR using primers specific for *LmCys2* (lane 1) and one isolate that did not (lane 2). All genomic DNA was digested with *Hind*III. For three isolates, a 300 bp size difference was observed for genomic DNA fragments hybridising to the *AvrLm6* probe. Since no *Hind*III sites are present in the coding sequence of *AvrLm6* the size difference must be due to polymorphisms in *Hind*III sites outside the region analysed.
(1.55 MB EPS)

Figure S2 Distribution of RIP mutations across the NC3 region, *LmCys1* and *LmTrans*. The ratio of mutated CpA or TpG sites was compared to the number of CpA and TpG nucleotides present in the wild type allele over a 100 bp window for the NC3 region (A) and *LmCys1* (B) and *LmTrans* (C) genes. Regions where greater than 50% of potential RIP sites are mutated are highlighted in grey. The gene structure of *LmCys1* and *LmTrans* are represented above the respective graphs.
(1.19 MB EPS)

Table S1 *Leptosphaeria maculans* isolates used in this study.
(0.04 MB DOC)

Table S2 Primers used in this study.
(0.06 MB DOC)

Table S3 Predicted gene structure of open reading frames within the *AvrLm1-LmCys2* genomic region of *Leptosphaeria maculans* isolate v23.1.3.
(0.03 MB DOC)

Table S4 Haplotype characterisation of 295 Australian isolates of *Leptosphaeria maculans* based on alleles of *AvrLm1, AvrLm6, LmCys1* and *LmCys2*.
(0.05 MB DOC)

Table S5 Alleles of *LmTrans, LmGT* and *LmMFS* in 84 Australian isolates of *Leptosphaeria maculans*.
(0.04 MB DOC)

Table S6 Haplotype characterisation of 84 Australian isolates of *Leptosphaeria maculans* based on alleles of seven genes and four non-coding, non-repetitive regions.
(0.11 MB DOC)

Table S7 Changes in allele frequencies of *AvrLm1, AvrLm6, LmCys1* and *LmCys2*.
(0.04 MB DOC)
