# Supplementary material for: Evolution of Linked Avirulence Effectors in Leptosphaeria maculans Is Affected by Genomic Environment and Exposure to Resistance Genes in Host Plants
Source: PLoS Pathog. 2010 Nov 4;6(11):e1001180. doi: 10.1371/journal.ppat.1001180 (PMC2973834; doi:10.1371/journal.ppat.1001180)
Supplement: Table S1 — Leptosphaeria maculans isolates used in this study. (0.04 MB DOC) [file ppat.1001180.s003.doc]

Table S1. *Leptosphaeria maculans* isolates used in this study

| Year^a^ | No. of isolates | Location^b^ (number of isolates) | Stubble source^d^ (number of isolates) |
| --- | --- | --- | --- |
| 1987 | 6 | Vic (6)^c^ | Polygenic (6) |
| 1988 | 13 | Vic (6) ^c^, SA (5), NSW (2) | Polygenic (13) |
| 1997 | 13 | Vic (13) | Polygenic (13) |
| 2001 | 15 | Vic (15) | Polygenic (13) |
| 2002 | 44 | Vic (21), SA (12), NSW (7), WA (4) | Polygenic (44) |
| 2003 | 46 | Vic (16), SA (6), NSW (15), WA (9) | Polygenic (46) |
| 2004 | 31 | Vic (4), SA (20), WA (7) | Polygenic (4 Vic, 11 SA, 5 WA),  Sylvestris (9 SA, 2 WA) |
| 2005 | 38 | Vic (4), SA (21), NSW (12), WA (1) | Polygenic (13 SA, 3 NSW),  Sylvestris (4 Vic, 8 SA, 9 NSW, 1 WA) |
| 2006 | 44 | Vic (19), SA (15), NSW (10) | Polygenic (8 Vic, 11 SA, 5 NSW),  Sylvestris (3 Vic, 4 SA, 5 NSW),  Juncea (8 Vic) |
| 2007 | 20 | Vic (20) | Juncea |
| 2008 | 25 | Vic (22), SA (3) | Polygenic (12 Vic, 1 SA),  Sylvestris (10 Vic),  Juncea (2 SA) |

^a^ Year which stubble was collected.

^b^ Victorian (Vic) sites included Beulah, Geelong, Horsham, Lake Bolac, Numurkah, Penshurst and Wonwondah. South Australian (SA) sites included Bool Lagoon, Bordertown, Edillilie, Millicent, Minlaton, Mundalla, Sherwood, Straun, Wolseley and Yeelana. New South Wales (NSW) sites included Galong, Grenfell, Illabo, Lockhart, Thuddungra and Wagga Wagga. West Australian (WA) sites included Mount Barker and Wongan Hills.

^c^ Includes historic Australian *L. maculans* isolates from the International Blackleg of Crucifers Network collection.

^d^ ‘Polygenic’ refers to Australian cultivars that are often heterogeneous for one of more resistance genes. ‘Sylvestris’ refers to Australian cultivars that contain major gene resistance derived from *B. rapa* subsp ‘*sylvestris’* [13]. ‘Juncea’ refers to Australian *B. juncea* cultivars.
