# Supplementary material for: Evolution of Linked Avirulence Effectors in Leptosphaeria maculans Is Affected by Genomic Environment and Exposure to Resistance Genes in Host Plants
Source: PLoS Pathog. 2010 Nov 4;6(11):e1001180. doi: 10.1371/journal.ppat.1001180 (PMC2973834; doi:10.1371/journal.ppat.1001180)
Supplement: Table S2 — Primers used in this study. (0.06 MB DOC) [file ppat.1001180.s004.doc]

Table S2. Primers used in this study

| Gene or  Region | Primer name ^b^ | Sequence (5’ to 3’) | Use^c^ |
| --- | --- | --- | --- |
| *AvrLm1* | AvrLm1-F | AATCCATTCCTCACCTCGTG | Seq, Probe |
|  | AvrLm1-R | GCACCAGAGGCAAAGACTTC |  |
|  | AvrLm1RT-F | CGCACTCTATACACACTCTA | RT-PCR |
|  | AvrLm1RT-R | GTCCGTTTCATGTGAGGCGAAGTAA |  |
| *AvrLm6* | AvrLm6-F | TCAATTTGTCTGTTCAAGTTATGGA | Seq, Probe |
|  | AvrLm6-R | CCAGTTTTGAACCGTAGAGGTAGCA |  |
|  | AvrLm6RT-F | AAACGGCACTATTACGAAAA | RT-PCR |
|  | AvrLm6RT-R | GATTAGGCGAGAAGCAAGT |  |
| *LmCys1*^a^ | LmCys1F | cagttcattggccttcgact | Seq, RT-PCR |
|  | LmCys1F | agtaactgtggcccttgctg |  |
| NC1^a^ | NC1-F | ATTGTGAGTACCCCCTGCTG | Seq |
|  | NC1-R | GCGACAGAAGACCAGAATCC |  |
| NC2^a^ | NC2-F | CCTTCCTCAAACACGCTCTC | Seq |
|  | NC2-R | GCCAATCTCTCCCGATATGA |  |
| NC3^a^ | NC3-F | AGCAATCCACCATTGGTCTC | Seq |
|  | NC3-R | TCGAAGCGCTAAGCCACTAT |  |
| NC4^a^ | NC4-F | CACGTGGACTTCAGCTACCA | Seq |
|  | NC4-R | AGGGCCTACGCTTAACGACT |  |
| *LmTrans*^a^ | LmTrans-F | CCCCACGAAAACCTGAACTA | Seq |
|  | LmTrans-R | TCACAGTCGAGTCGGACACT |  |
|  | LmTrans-int | TGTACAACTTTGCCGAGACG |  |
| *LmGT*^a^ | LmGT-F | ATCATGACTTGCTCGCTCAC | Seq |
|  | LmGT-R | TGGTAAAGAAGGCTCCCACA |  |
| *LmMFS*^a^ | LmMFS-1F | ACCATACAAATCGCGTCACA | Seq |
|  | LmMFS-1R | TTCCCGCTATAACGAACCAG |  |
|  | LmMFS-1int | GAGGCTGGTCTTGGTGTTTC |  |
|  | LmMFS-2F | CTGCCGAAGTGAGCATGTAG |  |
|  | LmMFS-2R | CTCCCGTCATCGAAAACACT |  |
| *LmCys2*^a^ | LmCys2-F | AACCAATGATCCTCCCATCTT | Seq, Probe |
|  | LmCys2-R | AGCGTCTAACGTAAGCAGACA |  |
|  | LmCys2RT-F | AACCAATGATCCTCCCATCTT | RT-PCR |
|  | LmCys2RT-R | AGCGTCTAACGTAAGCAGACA |  |
| Mating type locus | MAT1-1 | CTCGATGCAATGTACTTGG | PCR |
|  | MAT1-2 | AGCCGGAGGTGAAGTTGAAGCCG |  |
|  | MATidiom | TGGCGAATTAAGGGATTGCTG |  |
| actin | Act RT-F | AGTGCGATGTCGATGTCAG | RT-PCR |
|  | Act RT-R | AAGAGCGGTGATTTCCTTCT |  |

^a^ Genes and non-coding regions identified using BAC accessions (CT485669, CT485790, CT485667, CT485649 and CT485648).

^b^ Primers published previously. AvrLm1RT [9]; AvrLm6, AvrLm6RT [10], MAT [48] and ActRT [10].

^c^ Seq = Sequencing of alleles, Probe = generation of probes for southern analysis, RT-PCR = gene expression analysis *in planta*, PCR = PCR for mating-type locus.
