# Supplementary material for: Evolution of Linked Avirulence Effectors in Leptosphaeria maculans Is Affected by Genomic Environment and Exposure to Resistance Genes in Host Plants
Source: PLoS Pathog. 2010 Nov 4;6(11):e1001180. doi: 10.1371/journal.ppat.1001180 (PMC2973834; doi:10.1371/journal.ppat.1001180)
Supplement: Table S3 — Predicted gene structure of open reading frames within the AvrLm1-LmCys2 genomic region of Leptosphaeria maculans isolate v23.1.3. (0.03 MB DOC) [file ppat.1001180.s005.doc]

Table S3. Predicted gene structure of open reading frames within the *AvrLm1-LmCys2* genomic region of *Leptosphaeria maculans* isolate v23.1.3.

| Gene | Coding sequence (bp) | Protein  (amino acids) | Number of exons | Number of introns | Accession number |
| --- | --- | --- | --- | --- | --- |
| *AvrLm1* | 667 | 205 | 2 | 1 | AM084345 |
| *AvrLm6* | 581 | 144 | 4 | 3 | AM259336 |
| *LmCys1* | 663 | 220 | 1 | 0 | GU332625 |
| *LmTrans* | 1370 | 373 | 5 | 4 | GU332626 |
| *LmGT* | 1245 | 414 | 1 | 0 | GU332627 |
| *LmMFS* | 1852 | 591 | 2 | 1 | GU332628 |
| *LmCys2* | 774 | 247 | 1 | 0 | GU332629 |
