# Supplementary material for: Evolution of Linked Avirulence Effectors in Leptosphaeria maculans Is Affected by Genomic Environment and Exposure to Resistance Genes in Host Plants
Source: PLoS Pathog. 2010 Nov 4;6(11):e1001180. doi: 10.1371/journal.ppat.1001180 (PMC2973834; doi:10.1371/journal.ppat.1001180)
Supplement: Table S4 — Haplotype characterisation of 295 Australian isolates of Leptosphaeria maculans based on alleles of AvrLm1, AvrLm6, LmCys1 and LmCys2. (0.05 MB DOC) [file ppat.1001180.s006.doc]

Table S4. Haplotype characterisation of 295 Australian isolates of *Leptosphaeria maculans* based on alleles of *AvrLm1*, *AvrLm6,* *LmCys1* and *LmCys2*.

|  | Avirulence allele | | | | Number of isolates | Frequency (%) |
| --- | --- | --- | --- | --- | --- | --- |
| Haplotype | *AvrLm1* | *AvrLm6* | *LmCys1* | *LmCys2* |
| 1 | 0 | 0 | 0 | 0 | 10 | 3.4 |
| 2 | 0 | 0 | 1 | 0 | 42 | 14.2 |
| 3 | 0 | 1 | 0 | 0 | 16 | 5.5 |
| 4 | 0 | 1 | 1 | 0 | 82 | 27.8 |
| 5 | 0 | 3 | 1 | 0 | 2 | 0.7 |
| 6 | 0 | 4 | 3 | 0 | 2 | 0.7 |
| 7 | 0 | 9^a^ | 1 | 0 | 2 | 0.7 |
| 8 | 0 | 11^a^ | 1 | 0 | 2 | 0.7 |
| 9 | 0 | del | 0 | 0 | 2 | 0.7 |
| 10 | 0 | del | 1 | 0 | 24 | 8.1 |
| 11 | 1 | 0 | 1 | 0 | 1 | 0.3 |
| 12 | 1 | 1 | 1 | 0 | 2 | 0.7 |
| 13 | 1 | 5^a^ | 1 | 0 | 1 | 0.3 |
| 14 | 1 | del | 1 | 0 | 3 | 1.0 |
| 15 | 1 | del | 2 | del | 2 | 0.7 |
| 16 | 2 | del | 1 | 0 | 1 | 0.3 |
| 17 | 3 | 1 | 1 | 0 | 1 | 0.3 |
| 18 | 3 | del | 1 | 0 | 1 | 0.3 |
| 19 | 4 | 0 | 1 | 0 | 1 | 0.3 |
| 20 | del | 0 | 0 | 0 | 1 | 0.3 |
| 21 | del | 0 | 1 | 0 | 15 | 5.1 |
| 22 | del | 1 | 0 | 0 | 5 | 1.7 |
| 23 | del | 1 | 1 | 0 | 28 | 9.5 |
| 24 | del | 2 | 1 | 0 | 4 | 1.4 |
| 25 | del | 3 | 0 | 0 | 1 | 0.3 |
| 26 | del | 3 | 1 | 0 | 1 | 0.3 |
| 27 | del | 6^a^ | 1 | 0 | 1 | 0.3 |
| 28 | del | 7^a^ | 1 | 0 | 3 | 1.0 |
| 29 | del | 8^a^ | 1 | 0 | 2 | 0.7 |
| 30 | del | 8^a^ | 4^a^ | 0 | 1 | 0.3 |
| 31 | del | 9^a^ | 1 | 0 | 1 | 0.3 |
| 32 | del | 10^a^ | 1 | 0 | 1 | 0.3 |
| 33 | del | del | 0 | 0 | 17 | 5.9 |
| 34 | del | del | 1 | 0 | 17 | 5.9 |

^a^ RIP alleles
