# Supplementary material for: Evolution of Linked Avirulence Effectors in Leptosphaeria maculans Is Affected by Genomic Environment and Exposure to Resistance Genes in Host Plants
Source: PLoS Pathog. 2010 Nov 4;6(11):e1001180. doi: 10.1371/journal.ppat.1001180 (PMC2973834; doi:10.1371/journal.ppat.1001180)
Supplement: Table S5 — Alleles of LmTrans, LmGT and LmMFS in 84 Australian isolates of Leptosphaeria maculans. (0.04 MB DOC) [file ppat.1001180.s007.doc]

Table S5. Alleles of *LmTrans*, *LmGT* and *LmMFS* in 84 Australian isolates of *Leptosphaeria maculans*.

|  |  | Number of isolates  (frequency %) | Nucleotide changes^c^ | | | | Coding sequence changes^d^ | RIP dominance score^e^ |
| --- | --- | --- | --- | --- | --- | --- | --- | --- |
|  |  |  |  | Type | | |  |  |
| Gene^a,b^ | ‘Allele’ |  | No. | CpA to TpA | TpG to TpA | Other (Change) |  |  |
| *LmTrans* | 0 | 83 (98.8) | 0 | 0 | 0 | 0 |  | 0 |
|  | 1 | 1 (1.2) | 114 | 37 | 47 | 30 (all G to A or C to T) | 47 N-S, 16 SC | 14.0 |
| *LmGT* | 0 | 84 (100) | 0 | 0 | 0 | 0 |  | 0 |
| *LmMFS* | 0 | 79 (94.0) | 0 | 0 | 0 | 0 |  | 0 |
|  | 1 | 4 (4.8) | 1 | 0 | 0 | 1 (G-C) | Q^100^H | 0 |
|  | 2 | 1 (1.2) | 1 | 1 | 0 | 0 | SYN | 0 |

^a^ Reference sequences are CT485669 (designated as *LmTrans-0*), CT485669 (*LmGT-0*), CT485669 (*LmMFS-0*) identified from isolate v23.1.3 [9,10].

^b^ Sizes of the amplified products were 1783 bp for *LmTrans-0*, 1345 bp for *LmGT-0* and 1948 bp for *LmMFS-0*.

^c^ Deletion was confirmed by Southern analysis (Figure S1)

^d^ SYN, synonymous amino acid substitutions; N-S, non-synonymous substitutions; SC, premature stop codons

^e^ Allele sequences were analysed by RIPCAL for the presence of RIP mutations [24]. All sequences were compared to the wild-type allele (designated *-0*). RIP dominance scores of >1 are highly RIP-affected, whilst scores of 0 reflect the absence of RIP.
