# Supplementary material for: Evolution of Linked Avirulence Effectors in Leptosphaeria maculans Is Affected by Genomic Environment and Exposure to Resistance Genes in Host Plants
Source: PLoS Pathog. 2010 Nov 4;6(11):e1001180. doi: 10.1371/journal.ppat.1001180 (PMC2973834; doi:10.1371/journal.ppat.1001180)
Supplement: Table S6 — Haplotype characterisation of 84 Australian isolates of Leptosphaeria maculans based on alleles of seven genes and four non-coding, non-repetitive regions. (0.11 MB DOC) [file ppat.1001180.s008.doc]

Table S6. Haplotype characterisation of 84 Australian isolates of *Leptosphaeria maculans* based on alleles of seven genes and four non-coding, non-repetitive regions.

|  | Alleles of genes and of non-coding, non-repetitive regions | | | | | | | | | | | Number isolates | Frequency (%) |
| --- | --- | --- | --- | --- | --- | --- | --- | --- | --- | --- | --- | --- | --- |
| Haplotype | *AvrLm1* | NC1 | NC2 | NC3 | *AvrLm6* | *LmCys1* | NC4 | *LmTrans* | *LmGT* | *LmMFS* | *LmCys2* |
| 1 | 0 | 0 | 0 | 0 | 0 | 0 | 0 | 0 | 0 | 0 | 0 | 4 | 4.9 |
| 2 | 0 | 0 | 0 | 0 | 0 | 0 | 0 | 0 | 0 | 2 | 0 | 1 | 1.2 |
| 3 | 0 | 0 | 1 | 0 | 0 | 0 | 0 | 0 | 0 | 0 | 0 | 1 | 1.2 |
| 4 | 0 | 0 | 0 | 0 | 0 | 1 | 0 | 0 | 0 | 0 | 0 | 8 | 9.8 |
| 5 | 0 | 0 | 2 | 0 | 0 | 1 | 0 | 0 | 0 | 0 | 0 | 1 | 1.2 |
| 6 | 0 | 0 | 0 | 0 | 1 | 0 | 0 | 0 | 0 | 0 | 0 | 3 | 3.7 |
| 7 | 0 | 0 | 0 | 0 | 1 | 1 | 0 | 0 | 0 | 0 | 0 | 3 | 3.7 |
| 8 | 0 | 0 | 1 | 0 | 1 | 1 | 0 | 0 | 0 | 0 | 0 | 5 | 6.1 |
| 9 | 0 | 0 | 1 | 3 | 3 | 1 | 0 | 0 | 0 | 0 | 0 | 1 | 1.2 |
| 10 | 0 | 0 | 0 | 0 | 4 | 3 | 0 | 0 | 0 | 0 | 0 | 2 | 2.4 |
| 11 | 0 | 0 | 1 | 8 ^a^ | 9^a^ | 1 | 0 | 0 | 0 | 0 | 0 | 1 | 1.2 |
| 12 | 0 | 0 | 1 | 10 ^a^ | 9 ^a^ | 1 | 0 | 0 | 0 | 0 | 0 | 1 | 1.2 |
| 13 | 0 | 0 | 1 | 11 ^a^ | 11 ^a^ | 1 | 0 | 0 | 0 | 0 | 0 | 2 | 2.4 |
| 14 | 0 | 0 | 0 | 0 | del | 0 | 0 | 0 | 0 | 0 | 0 | 1 | 1.2 |
| 15 | 0 | 0 | 0 | 0 | del | 1 | 0 | 0 | 0 | 0 | 0 | 1 | 1.2 |
| 16 | 0 | 0 | 0 | 1 | del | 1 | 0 | 0 | 0 | 0 | 0 | 1 | 1.2 |
| 17 | 1 | 0 | 1 | 0 | 0 | 1 | 0 | 0 | 0 | 0 | 0 | 1 | 1.2 |
| 18 | 1 | 0 | 0 | 0 | 1 | 1 | 0 | 0 | 0 | 0 | 0 | 1 | 1.2 |
| 19 | 1 | 0 | 1 | 9 ^a^ | 5 ^a^ | 1 | 0 | 0 | 0 | 0 | 0 | 1 | 1.2 |
| 20 | 1 | 0 | 0 | 0 | del | 1 | 0 | 0 | 0 | 0 | 0 | 2 | 2.4 |
| 21 | 1 | 0 | 1 | 0 | del | 2 | 0 | 0 | 0 | 1 | del | 1 | 1.2 |
| 22 | 2 | 0 | 1 | 0 | del | 1 | 0 | 0 | 0 | 0 | 0 | 1 | 1.2 |
| 23 | 3 | 0 | 0 | 0 | 1 | 1 | 0 | 0 | 0 | 0 | 0 | 1 | 1.2 |
| 24 | del | 2 | 1 | 0 | 0 | 0 | 0 | 0 | 0 | 0 | 0 | 1 | 1.2 |
| 25 | del | 0 | 0 | 0 | 0 | 1 | 0 | 0 | 0 | 0 | 0 | 2 | 2.4 |
| 26 | del | 2 | 0 | 0 | 0 | 1 | 0 | 0 | 0 | 0 | 0 | 2 | 2.4 |
| 27 | del | 2 | 1 | 0 | 0 | 1 | 0 | 0 | 0 | 0 | 0 | 1 | 1.2 |
| 28 | del | 0 | 0 | 0 | 1 | 0 | 0 | 0 | 0 | 0 | 0 | 1 | 1.2 |
| 29 | del | 2 | 3 | 0 | 1 | 0 | 0 | 0 | 0 | 0 | 0 | 3 | 3.7 |
| 30 | del | 0 | 0 | 0 | 1 | 1 | 0 | 0 | 0 | 0 | 0 | 1 | 1.2 |
| 31 | del | 0 | 1 | 0 | 1 | 1 | 0 | 0 | 0 | 0 | 0 | 1 | 1.2 |
| 32 | del | 0 | 1 | 2 | 1 | 1 | 0 | 0 | 0 | 0 | 0 | 1 | 1.2 |
| 33 | del | 2 | 0 | 0 | 1 | 1 | 0 | 0 | 0 | 0 | 0 | 1 | 1.2 |
| 34 | del | 0 | 1 | 0 | 2 | 1 | 0 | 0 | 0 | 1 | 0 | 3 | 3.7 |
| 35 | del | 1 | 1 | 0 | 2 | 1 | 0 | 0 | 0 | 1 | 0 | 1 | 1.2 |
| 36 | del | 0 | 0 | 12 ^a^ | 6 ^a^ | 1 | 0 | 0 | 0 | 0 | 0 | 1 | 1.2 |
| 37 | del | 0 | 0 | 5 ^a^ | 7 ^a^ | 1 | 0 | 0 | 0 | 0 | 0 | 1 | 1.2 |
| 38 | del | 0 | 1 | 12 ^a^ | 7 ^a^ | 1 | 0 | 0 | 0 | 0 | 0 | 1 | 1.2 |
| 39 | del | 2 | 0 | 12 ^a^ | 7 ^a^ | 1 | 0 | 0 | 0 | 0 | 0 | 1 | 1.2 |
| 40 | del | 0 | 0 | 4 ^a^ | 8 ^a^ | 1 | 0 | 0 | 0 | 0 | 0 | 1 | 1.2 |
| 41 | del | 2 | 0 | 4 ^a^ | 8 ^a^ | 1 | 0 | 0 | 0 | 0 | 0 | 1 | 1.2 |
| 42^b^ | del | 0 | 0 | 6 ^a^ | 8 ^a^ | 4 ^a^ | 1 ^a^ | 1 ^a^ | 0 | 0 | 0 | 1 | 1.2 |
| 43 | del | 2 | 0 | 7 ^a^ | 9 ^a^ | 1 | 0 | 0 | 0 | 0 | 0 | 1 | 1.2 |
| 44 | del | 2 | 0 | 13 ^a^ | 10 ^a^ | 1 | 0 | 0 | 0 | 0 | 0 | 1 | 1.2 |
| 45 | del | 0 | 0 | 0 | del | 0 | 0 | 0 | 0 | 0 | 0 | 2 | 2.4 |
| 46 | del | 0 | 1 | 0 | del | 0 | 0 | 0 | 0 | 0 | 0 | 1 | 1.2 |
| 47 | del | 2 | 0 | 0 | del | 0 | 0 | 0 | 0 | 0 | 0 | 1 | 1.2 |
| 48 | del | 2 | 1 | 0 | del | 0 | 0 | 0 | 0 | 0 | 0 | 1 | 1.2 |
| 49 | del | 0 | 0 | 0 | del | 1 | 0 | 0 | 0 | 0 | 0 | 1 | 1.2 |
| 50 | del | 0 | 1 | 0 | del | 1 | 0 | 0 | 0 | 0 | 0 | 1 | 1.2 |
| 51 | del | 2 | 0 | 0 | del | 1 | 0 | 0 | 0 | 0 | 0 | 4 | 4.9 |

^a^ RIP alleles

^b^ Haplotype 42 has RIP alleles at five loci
