# Supplementary material for: Evolution of Linked Avirulence Effectors in Leptosphaeria maculans Is Affected by Genomic Environment and Exposure to Resistance Genes in Host Plants
Source: PLoS Pathog. 2010 Nov 4;6(11):e1001180. doi: 10.1371/journal.ppat.1001180 (PMC2973834; doi:10.1371/journal.ppat.1001180)
Supplement: Table S7 — Changes in allele frequencies of AvrLm1, AvrLm6, LmCys1 and LmCys2. (0.04 MB DOC) [file ppat.1001180.s009.doc]

Table S7. Changes in allele frequencies of *AvrLm1*, *AvrLm6,* *LmCys1* and *LmCys2*.

| Gene | Allele | Number of isolates (frequency %) | |
| --- | --- | --- | --- |
|  |  | 1987 – 2003 | 2004 -2008 |
| *AvrLm1* | 0 | 100 (73.0) | 85 (53.8) |
|  | 1 | 3 (2.2) | 6 (3.8) |
|  | 2 | 0 (0) | 1 (0.6) |
|  | 3 | 2 (1.5) | 0 (0) |
|  | 4 | 1 (0.7) | 0 (0) |
|  | del | 31 (22.6) | 66 (41.8) |
| *AvrLm6* | 0 | 43 (31.3) | 27 (17.1) |
|  | 1 | 82 (59.9) | 52 (32.9) |
|  | 2 | 0 (0) | 4 (2.5) |
|  | 3 | 4 (2.9) | 0 (0) |
|  | 4 | 2 (1.5) | 0 (0) |
|  | 5^a^ | 0 (0) | 1 (0.6) |
|  | 6^a^ | 0 (0) | 1 (0.6) |
|  | 7^a^ | 0 (0) | 3 (1.9) |
|  | 8^a^ | 0 (0) | 3 (1.9) |
|  | 9^a^ | 0 (0) | 3 (1.9) |
|  | 10^a^ | 0 (0) | 1 (0.6) |
|  | 11^a^ | 0 (0) | 2 (1.3) |
|  | del | 6 (4.4) | 61 (38.7) |
| *LmCys1* | 0 | 21 (15.3) | 31 (19.6) |
|  | 1 | 112 (81.7) | 126 (79.8) |
|  | 2 | 2 (1.5) | 0 (0) |
|  | 3 | 2 (1.5) | 0 (0) |
|  | 4^a^ | 0 (0) | 1 (0.6) |
| *LmCys2* | 0 | 135 (98.5) | 158 (100) |
|  | del | 2 (1.5) | 0 (0) |

^a^ alleles associated with RIP
